# Dynamical efficiency for multimodal time-varying transportation networks

**DOI:** 10.1038/s41598-021-02418-5

**Published:** 2021-11-29

**Authors:** Leonardo Bellocchi, Vito Latora, Nikolas Geroliminis

**Affiliations:** 1 Urban Transport Systems Laboratory (LUTS), École Polytechnique Fédérale de Lausanne (EPFL), GC C2 390, Station 18, Lausanne, 1015 Switzerland; 2 School of Mathematics, Queen Mary University of London (QMUL), E5 Mile Road, London, UK; 3 Dipartimento di Fisica ed Astronomia, Università di Catania and INFN, 95123 Catania, Italy

**Keywords:** Complex networks, Civil engineering

## Abstract

Spatial systems that experience congestion can be modeled as weighted networks whose weights dynamically change over time with the redistribution of flows. This is particularly true for urban transportation networks. The aim of this work is to find appropriate network measures that are able to detect critical zones for traffic congestion and bottlenecks in a transportation system. We propose for both single and multi-layered networks a path-based measure, called dynamical efficiency, which computes the travel time differences under congested and free-flow conditions. The dynamical efficiency quantifies the reachability of a location embedded in the whole urban traffic condition, in lieu of a myopic description based on the average speed of single road segments. In this way, we are able to detect the formation of congestion seeds and visualize their evolution in time as well-defined clusters. Moreover, the extension to multilayer networks allows us to introduce a novel measure of centrality, which estimates the expected usage of inter-modal junctions between two different transportation means. Finally, we define the so-called dilemma factor in terms of number of alternatives that an interconnected transportation system offers to the travelers in exchange for a small increase in travel time. We find macroscopic relations between the percentage of extra-time, number of alternatives and level of congestion, useful to quantify the richness of trip choices that a city offers. As an illustrative example, we show how our methods work to study the real network of a megacity with probe traffic data.

## Introduction

Analyzing urban mobility is one of the fundamental tasks of traffic engineering. This is due to the enormous consequences that efficient or inefficient transportation system can have on all citizens’ lifes not only in terms of travel time but also of pollution, stress, safety, public expenses. In modern cities, urban traffic is represented by an integrated and interconnected system that includes private cars but also public transportation like the underground, buses, car and/or bike-sharing system, etc.. This system of systems cannot be understood and properly managed by decomposing its components’ properties, as it is governed by non-linear interactions of entities with various objectives in a limited urban space. The new era of big data has raised our expectations to make mobility more predictable and controllable through a better utilization of existing resources and capacity. Capacity has many different dimensions in transport systems, as it can refer to roads, vehicles or networks, all in a time-dependent manner. The influence of local bottlenecks at the network level is still unclear, despite recent efforts in network modeling (see for example Refs.^1–6^), but we have now the possibility to exploit real-time information and not fine-grained dynamic equilibrium models (that require a large number of parameters and are difficult to calibrate) in order to understand the network criticalities and how they change overtime. A key challenge here is to define appropriate measures to quantify the performance of integrated multimodal urban transportation systems.

In the literature, especially in the domain of the complex networks studies, the analysis of the network performance affected by congestion has been mostly studied with a percolation approach^7–12^, considering a congested road inaccessible when the average speed in that segment goes below a certain threshold. However, each road can contain large numbers of vehicles, and the service time required for such vehicles to reach their destinations is not taken into account. Interesting results about the robustness of the transportation network and the disruption of the main connected component come from the results in Ref.^13^, where the authors identify the critical links for the mobility in real urban networks of different cities. Other studies suggest that also the functional and the geometrical topology of the transportation network influence the congestion propagation and they use structural information to recognize traffic hotspots (for example^14,15^). Moreover, concepts as simplicity^16^ and straightness centrality^17^ have been correlated with the navigation and the drivers’ behaviour, and in ultimate analysis with the network response to a severe traffic demand^18–20^. The accessibility of huge urban sensing data permit to obtain detailed information on human mobility, and then to calibrate models and networks analysis in urban transportation as done, for instance in Refs.^19–23^. Another interesting study on the evaluation of transportation networks at different spatial scales using information on traffic pattern and on stations topology has been conducted in Ref.^24^.

In the last decades, there has been a large interest in the analysis of multimodal transportation systems and in the optimization of traffic flows in systems with interdependent public and private mobility. Reference^25^ has approached the problem of finding optimized algorithms for the computation of shortest paths in multimodal environments, while the authors of Ref.^26^ have empirically investigated the interconnectivity between different modes of transportation in large data sets of real trip choices. A theory of maximum traffic flow for co-existing transportation modes based on the relation among accumulation, speed and flow has been introduced in Ref.^27^, together with the concept of the three-dimensional Macroscopic Fundamental Diagram (3D-MFD), and has been applied to analyze real case studies as in Ref.^28^. MFD gathers together and measures the network effects of traffic on a region of the city. Empirical observations^29,30^ have shown that by spatially aggregating the noisy plots of flow vs. density from individual links (known as Fundamental Diagram), the scatter decreases and a well-defined curve exists between space-mean flow and density. The shape of MFD is a property of the network topology and traffic control operations, while it is also influenced by the spatial heterogeneity of congestion. MFD can also be utilized to introduce elegant perimeter control or pricing strategies to improve mobility in multi-region and multi-modal networks, like in Refs.^31,32^, and others. These findings are of great importance because the concept of an MFD can be applied for heterogeneously loaded cities with multiple centers of congestion. Nevertheless, we remark that all network structure and node-to-node path choice is lost due to this spatial aggregation, meaning that the granularity of the aggregation does not allow to identify critical links in the network.

In this work we propose a general framework to characterize the interplay between the topology of a transportation network and the dynamical patterns of traffic demands and congestion. Such a framework allows a global analysis of an entire mobility system, identifies the formation of congestion seeds, and visualizes their evolution in time. In multimodal transportation networks, our method allows to introduce a novel measure of centrality, which estimates the expected usage of inter-modal junctions, i.e. of those junctions connecting two different transportation modes.

Crucitti et al. in Ref.^17^ have defined a concept of network efficiency by comparing the shortest path distance $d_{ij}$ between nodes *i* and *j* to their Euclidean distance, without considering traffic demands from *i* to *j*. Later, Nagurney et al.^33^ have defined a new measure of efficiency that, instead of the distance $d_{ij}$, considers the equilibrium travel time $\lambda _{ij}$ from *i* to *j*, weighted by the demand $\delta _{ij}$ of this specific Origin-Destination pair. They also proposed a pseudo dynamic version of this measure which is an overall average performance from time 0 to *T* (e.g. the whole duration of a simulation or a whole day) where $\lambda _{ij}$ and $\delta _{ij}$ vary across time. The quantity $\lambda _{ij}$ is defined according to Wardrop’s principle, where the cost of all used routes between *i* and *j* are the same, while the rest is higher. This is equivalent to that all trips from *i* to *j* travel through the shortest travel-time path.

In our work, inspired both by Refs.^17,33^, we define the *dynamical efficiency* as a real dynamical measure that changes every short period (e.g. 5–15 min). Such a measure allows to see how congestion development during the peak periods influences the efficiency of the network. We also investigate how the spatial distribution of node efficiency at the local level can help to identify the critical nodes of a network. Interestingly, we observe that nodes with high efficiency during uncongested conditions may have very different values when the network gets congested. This detailed consideration of efficiency in a finer spatio-temporal resolution allows to identify node criticalities and relate this with traffic congestion in a way that was not possible with the existing definitions of other network measures, as betweenness and others. Thus, we propose to utilize the efficiency not to evaluate the average performance of networks, but to integrate it in the dynamic framework of congestion and possibly create a new metric to trigger decisions for real-time traffic management. In our work we do not estimate travel times based on equilibrium models, but we receive this information from real-time GPS data. Then, we estimate shortest travel-time paths in the network based on the real link travel times. In such a way, the main objectives we achieve are: (i) to describe how traffic influences the level of performance of a multimodal transportation network considering realistic human behaviour (limited number of changes and choices) at local level; (ii) to obtain link efficiency values spatially smoothed that highlight congested zones and their gradual propagation in the spatial network; (iii) to estimate the state of a link based on its communication with the whole transportation network system and not as independent entity; (iv) to compute the ratio of the users’ travel time flexibility over the number of alternatives gained as the congestion grows and establish the level of multimodal richness of urban transportation system.

The paper is organized as follows. In the results section we introduce our centrality measures for single- and multi-layer transport systems. We also propose an efficient algorithm to compute shortest path in multiplex networks with constraints on the number and type of layer changes. We then devote an entire section of the article to show a practical application of our framework to the case of Shenzhen, a megacity in China for which GPS taxis traces and operational data from the underground metro are available.

**Figure 1:**
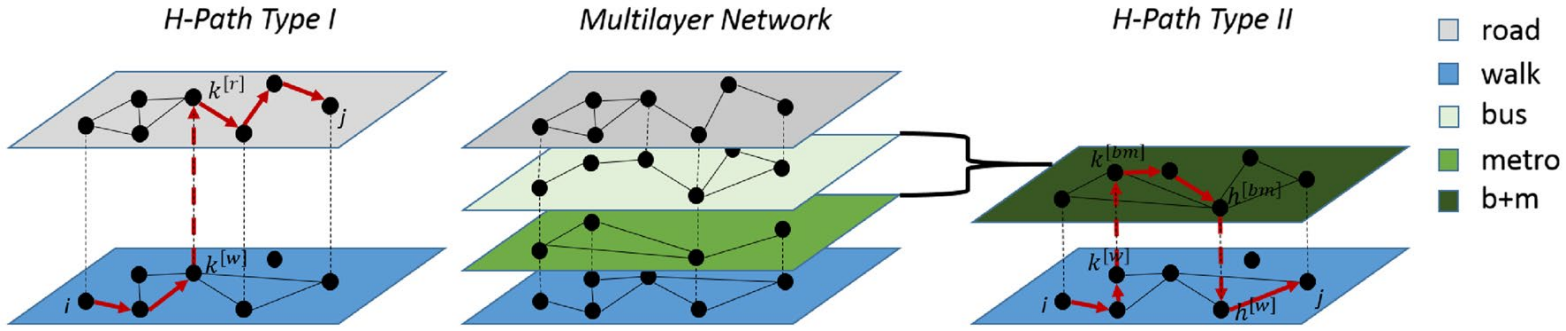
H-paths of type I and II in multimodal transportation networks. A transportation system is represented in the central panel as a multilayer network, where each layer accounts for a different mode of transportation, e.g, private cars (road), walking, bus system, metro. The left panel shows in red an example of a shortest H-path of *type I* with source and destination at two different layers and only one change at station *k*. The right panel is an example of a shortest H-path of *type II*. In this case the user moves to layer $[\mathrm{bm}]$ and travels in this layer from node $k^{[\mathrm{bm}]}$ to node $h^{[\mathrm{bm}]}$, and then continues her trip in the original layer until the final destination $j^{[\mathrm{w}]}$ is reached. As shown in this example, a layer can also be constructed by the aggregation of 2 or more layers (for example bus+metro $[\mathrm{bm}]$).

## Results

### Dynamical efficiency in a single layer

We describe a transportation system at time step *t* as a weighted graph ${\mathscr {G}}(t)= G({\mathscr{N}},{\mathscr{L}},{\mathscr{T}}(t))$, where ${\mathscr {N}} = \{n_1,n_2,\ldots ,n_N\}$ is the set of nodes, ${\mathscr {L}} = \{l_1,l_2,\ldots ,l_{L}\}$ is the set of links and ${\mathscr {T}} = \{\tau _1(t), \tau _2(t),\ldots , \tau _L(t)\}$ is a set of positive real numbers associated to the links. The number of nodes $N = | {\mathscr {N}}|$ and links $L = | {\mathscr {L}}|$ is fixed and we assume that topology of the network does not change in time, i.e. nodes and links cannot be created or removed, while the link weights can take different values at different time steps *t*, depending on the traffic conditions. The weight $\tau _{ij}(t)$ at time step *t* of link $\ell _{(i,j)}=(i,j)$ connecting node *i* to node *j* represents the travel time associated to traverse the link at a given temporal state *t* of the system. For each time step *t*, we have $\tau _{ij}(t) \ge \tau _{ij}$, where $\tau _{ij}$ is the travel time at free flow speed between *i* and *j*. Knowing the weights of all the links, at each given time step *t* it is possible to construct the path with the shortest possible travel time from any source node *i* to any destination node *h*, and therefore to define the travel time $\tau _{ih}(t)$ from *i* to *h*. Even in this case $\tau _{ih}(t) \ge \tau _{ih} \forall t$ where $\tau _{ih}$ is the minimum experienced travel time at free flow speed. Paths and travel times changes dynamically with the traffic, depending on the weights that network has at time step *t*. Then, for each link $\ell _{(i,j)}$ and time *t* we can define the *dynamical efficiency of the link* as: $E(\ell _{(i,j)} (t)) = \frac{e_i(t) + e_j(t)}{2}$, where

1 $$\begin{aligned} e_i(t) = \frac{1}{|N|-1}\sum _{h \in {\mathscr {N}}\setminus \{i\}}{\frac{\tau _{ih}}{\tau _{ih}(t)}}. \end{aligned}$$

The dynamical efficiency measures the ‘reachability’ of a link, which changes with the degree of network congestion. This quantity has the nice property of being spatially smoothed, and this is useful for traffic engineering to visualize the congested zones of a spatial network, without the need of any clustering algorithm^2,34^, and to apply congestion mitigation strategies, e.g. perimeter control as in Refs.^32,35,36^). By definition $E(\ell (t))$ takes values that range between 0 and 1. If $E(\ell (t)) \approx 0$, then link $\ell$ is almost inaccessible because the travel time to reach $\ell$ from other locations in network is much longer than in free flow condition. In the other extreme case, when $E(\ell (t)) \approx 1$, for each node of the network there is a path connecting link $\ell$ to the node with the minimum travel time. The *dynamical efficiency of the network* $E({\mathscr {G}}(t))$ can finally be defined as an average over the efficiency of all its links at time step *t*, $E({\mathscr {G}}(t)) = \frac{1}{L}\sum _{(\ell \in {\mathscr {L}})}E(\ell (t))$, and it is as well a quantity ranging in [0, 1]. What in the standard definition of network efficiency proposed in Ref.^17^ was the Euclidian distance between two points in the network is here replaced by the shortest time path in free-flow condition, while the length of the shortest path is replaced by the shortest time at a given state *t* of the network. This metric is similar to the static definition proposed in Ref.^33^, but include dynamic information. We will also show in the paper the importance of the evolution of link level efficiency $E_i$ in understanding congestion propagation and network criticalities. Another quantity that, using the introduced definition of travel times between two nodes, can be generalized to a dynamical case, is that of edge betweenness^17,37^. In the standard definition this is equal to the proportion of shortest paths containing a given link . Here, we can define the *dynamical betweenness centrality* $B(\ell (t))$ of link $\ell \in {\mathscr {L}}$ at time *t* as:

2 $$\begin{aligned} B(\ell (t)) = \frac{1}{N(N-1)} \sum _{i,j \in {\mathscr {N}}, i\ne j }\frac{\nu _{ij}(\ell (t))}{\nu _{ij}(t)} \end{aligned}$$

where $\nu _{ij}(t)$ and $\nu _{ij}(\ell (t))$ are respectively the total number of shortest time paths from node *i* to node *j* at time *t* and those among them which pass through link $\ell$. While in the standard definition of betweenness centrality (without the effect of congestion), links with the highest values of betweenness are located in the geometrical center of the network^38–40^, in congested transportation networks the dynamical betweenness takes into account the ‘spatial’ centrality of the links but also their contribution to the whole network performance in terms of travel time featuring, in this way, the fastest arterial roads but also the most critical ones for traffic load. This opens up new opportunities to study the evolution of urban congestion in terms of edge centralities of the network.

### Dynamical efficiency in multilayered networks

Let us now extend our argument to the case of multimodal transportation systems. This can now be modeled as a weighted graph with many layers^41,42^ ${\mathscr {M}}(t)$ whose weights change over time. If we indicate with *M* the number of layers, each layer *m*, with $m=1,2,\ldots ,M$, is described at time *t* by the weighted graph ${\mathscr {G}}^{[m]}(t)= G({\mathscr {N}}^{[m]},{\mathscr {L}}^{[m]},{\mathscr {T}}^{[m]}(t))$, where ${\mathscr {N}}^{[m]}, {\mathscr {L}}^{[m]}$ and ${\mathscr {T}}^{[m]}(t)$ are respectively the set of nodes, links and weights at layer *m*, and $N^{[m]} = | {\mathscr {N}}^{[m]}|$ and $L^{[m]} = | {\mathscr {L}}^{[m]}|$ are the numbers of nodes and links at layer *m*. Notice that even in this case the topology of each layer is fixed while the links weights can change in time depending on the traffic conditions. The links in ${\mathscr {L}}^{[m]}$ are called intralayer links. In order to fully specify a multimodal transportation system, we also need to define the links $\ell _{(k^{[m]}h^{[n]})}$ connecting a pair of nodes $k^{[m]} \in {\mathscr {N}}^{[m]}$ and $h^{[n]} \in {\mathscr {N}}^{[n]}$ of two different layers *m* and *n*, which are are called interlayer links. In our case study, we will consider only *multiplex networks*, a particular type of networks with many layers where all the interlayer links $\ell$ connect only the same node *k* in the different layers, that is $\ell _{(k^{[m]}k^{[n]})}$ with $k \in {\mathscr {K}}^{[m,n]} \equiv {\mathscr {N}}^{[m]} \cap {\mathscr {N}}^{[n]}$. Nevertheless, all our results can be easily extended to the more general case of multilayer networks. Finally, we define a *station* as an extreme point *k* of an interlayer link $\ell _{(k^{[m]}k^{[n]})}$ and $K^{[m,n]} = |{\mathscr {K}}^{[m,n]}|$ the number of stations between two layers ${\mathscr {G}}^{[m]}$ and ${\mathscr {G}}^{[n]}$.

The multilayer networks have been used in several domains since their first appearance in literature^43–45^. In particular, urban multimodal transportation systems can be easily represented by a multilayered network where each layer represents the topology and the characteristics of each single system (private cars, metro, bus, car- and bike-sharing, etc.) and where the interlayer links stand for the interchange stations that bridge one system to another.

#### The shortest H-path

Computing shortest paths in multilayered networks requires, in general, a huge computational effort due to the higher complexity with respect to a single layer network. Nevertheless, the fact that a traveler changes from one layer to another, with no limitation on the number of swaps, to follow the fastest path to his/her destination is not realistic. Therefore, we assume that in the transportation environment the most realistic paths are of 2 types^46^: (*I*) with only a change between one layer to another; or (*II*) with two opposite changes from a layer ${\mathscr {G}}^{[m]}$ to ${\mathscr {G}}^{[n]}$ by passing through two different interchange stations $h \ne k \in {\mathscr {K}}^{[m,n]}$. Figure 1 shows an example of *type* *I* and *II*. We call a path with these characteristics *H-path*. This strong but consistent assumption does not only take into consideration the peculiar properties of urban spatial paths but also, thanks to a computationally fast algebraic algorithm, it allows to drastically reduce the calculation cost. We show that for computing the shortest H-paths at any time *t* between a node *i* and a node *j* using two layers ${\mathscr {G}}^{[m]}(t)$ and ${\mathscr {G}}^{[n]}(t)$, we just need to run the all shortest paths algorithm individually for each layer and then sum the *i*-row of the node-to-node distance matrix of ${\mathscr {G}}^{[m]}(t)$ and the *j*-column of the corresponding distance matrix of ${\mathscr {G}}^{[n]}(t)$ to obtain the vector $\mathbf{v }^{[m,n]}_{ij}(t) = \{v^{[m,n]}_{ij,k}(t)\}_{k \in K^{[m,n]}}$ of travel times from $i \in {\mathscr {N}}^{[m]}(t)$ to $j \in {\mathscr {N}}^{[n]}(t)$ changing in station *k*. The minimum value $H^{[m,n]}_{ij}(t) =v^{[m,n]}_{ij,k^*}(t) = min\left\{ v^{[m,n]}_{ij,k}(t) = \tau ^{[m]}_{ik}(t)+ \tau ^{[n]}_{kj}(t)\right\} _{k \in {\mathscr {K}}^{[m,n]}}$ will be the travel time of the shortest H-path between *i* and *j* at time *t*, and $k^* (\ne i , j)$ the most convenient interchange station. A more detailed explanation of the algorithm has been reported in SI (Fig. S2).

An example of the H-path *type-I* is when a trip is made by car for the first part and for the rest by public transportation, while *type-II* represents the case when a traveller walks from an origin location to a metro station, takes the metro to move to another station and arrives at his/her destination by walking again. As in the example shown in Fig. 1, we can consider a layer as the aggregation of different single transportation networks as the *ensemble* of public transportation and then, in a second moment, calculate the shortest H-paths between this aggregated one and another layer of the transportation system. As shown in Fig. S3 of the SI, the complexity of this algorithm grows linearly with the number of layers *M*, while the complexity of an all-shortest-path algorithm in a multiplex network grows as a function of *M* as a power law with an exponent larger than 2. This algorithm can be extended allowing a larger number of transfers but this is beyond the scope of this work (as the majority of trips are of *type-I* or *II*).

#### Extended formula of dynamical efficiency

In a multilayer transportation network, we use the definition of shortest H-paths in duplex to compute the multilayer dynamical efficiency. We denote $H_{ij}^{[m,n]}(t)$ the time of the shortest H-path between *i* and *j* using layer *m* and *n* at time *t* and $H^*_{ij}(t) = H_{ij}^{[m^*,n^*]}(t) \left( \le H_{ij}^{[m,n]}(t), \ \forall m,n \le M\right)$ the shortest H-path of the whole network realized by the duplex $[m^*,n^*]$. Keeping the notation in Eq. (1), we define $H^*_{ij}(t)\ge \tau ^{[{\mathscr {M}}]}_{ij}$, for all time *t*, the shortest H-path in a free flow condition in the multiplex ${\mathscr {M}}$.

The *duplex dynamical efficiency for a link* $\ell ~\in ~{\mathscr {L}}^{[m,n]}$ is defined as $E^{[m,n]}(\ell _{(i,j)}(t)) = \frac{e^{[m,n]}_i(t) + e^{[m,n]}_j(t)}{2}$, where

3 $$\begin{aligned} e^{[m,n]}_i(t) = \frac{1}{N^{[m,n]}-1}\sum _{p \in {\mathscr {N}}^{[m,n]}\setminus \{i\}}{\frac{\tau ^{[{\mathscr {M}}]}_{ip}}{H^{[m,n]}_{ip}(t)}}, \end{aligned}$$

and $N^{[m,n]} = |{\mathscr {N}}^{[m,n]} |= |{\mathscr {N}}^{[m]} \cup {\mathscr {N}}^{[n]}|$, while the *multiplex dynamical efficiency* as $E(\ell _{(i,j)}(t)) = \frac{e_i(t) + e_j(t)}{2}$, where

4 $$\begin{aligned} e_i(t)= \frac{1}{N^{[{\mathscr {M}}]}-1}\sum _{q \in {\mathscr {N}}^{[{\mathscr {M}}]}\setminus \{i\}}{\frac{\tau ^{[{\mathscr {M}}]}_{iq}}{H^*_{iq}(t)}}, \end{aligned}$$

with $$\displaystyle N^{[{\mathscr {M}}]} = |{\mathscr {N}}^{[{\mathscr {M}}]} | = \left| \bigcup _{m= 1, 2,\dots , M} {\mathscr {N}}^{[m]}\right| $$.

### Layer performance analysis and station centrality

By comparing the efficiency of the entire multiplex ${\mathscr {M}}(t)$ with the efficiency of ${\mathscr {M}}^{[\setminus m]}(t)) = \bigcup _{n \ne m, n \le M}{\mathscr {G}}^{[n]}(t))$, that is ${\mathscr {M}}(t)$ without layer ${\mathscr {G}}^{[m]}$, we define the *dynamical layer gain* as

5 $$\begin{aligned} LG(m(t)) = \frac{E({\mathscr {M}}^{[\setminus m]}(t))}{E({\mathscr {M}}(t))}. \end{aligned}$$

This represents a powerful analysis tool to quantify the contribution of each layer to the whole mobility and a first step to calculate the cost/benefits of a transportation network component by component. For example, it can highlight the advantages in mobility by adding a number of bike sharing stations in critical locations of a city. Another important measure is the *station centrality*

6 $$\begin{aligned} \small { I^{[m,n]}(k(t)) = \frac{1}{N^{[m,n]}(N^{[m,n]}-1)}\sum _{i,j \in {\mathscr {N}}^{[m,n]}\setminus \{k\}} \delta _k^{k^*}\langle i,j, [m,n] \rangle (t)} \end{aligned}$$

where $\delta _k^{k*}\langle i,j, [m,n]\rangle (t) = 1$ if the shortest H-path between *i* and *j* goes through station *k* from layer *m* to layer *n* (i.e. $k=k^*$ and $[m,n] = [m^*,n^*]$) at time *t* and 0 otherwise. This measure is an indicator of how many times a station *k* has been used as interlayer junction by the all shortest H-paths algorithm. The station centrality is highly dependent on our original definition of shortest H-path and it can be seen, in a more general point of view, as the betweenness of the interlayer links considering one (*type-I*) or two opposite changes (*type-II*) for the shortest paths. Interestingly, stations with high betweenness based on the network topology can have low centrality when a layer of the network is highly congested.

### The $\alpha$ and $\beta$ dilemma factors

While travelers have the tendency to select paths that minimize their own travel time in a selfish way, there might be alternatives with slightly larger travel time that, if selected, can enhance the mobility of the entire system. This flexibility in terms of choice of routes with similar travel times under congested conditions could be an important practical tool for operators to decrease the price of anarchy and push the system to solutions closer to the system optimum^47^.

For each couple $(i,j)~\in ~ {\mathscr {N}}^{[m]}~\times ~ {\mathscr {N}}^{[n]}$ and time *t*, we are able to determine the rank of convenience of each interchange station $k \in {\mathscr {K}}^{[m,n]}$ by ordering the values of travel time in vector $\mathbf{v }^{[m,n]}_{ij}(t)$. Therefore, we can establish a measure of replacement of each station *k*, that we called *dilemma factor* and it tells us whether some other alternative H-paths, with respect to the fastest, are still convenient or not. The *dilemma* comes from the following question: is it better to have faster but non-replaceable paths or to have a larger number of alternative paths that are slightly slower than the fastest one? And, how much does it cost to users in terms of extra travel time to have more valid alternatives in a multimodal transportation system?

For the sake of clarity, in the following definitions whenever there is no ambiguity, we will omit the apex $$^{[m,n]}$$, being clear that all measures refer to shortest H-paths computed on a couple of layers [*m*, *n*] of the multiplex $${\mathscr {M}}(t)$$. For each trip from node *i* to node *j*, we define $$v_{ij,k^*}(t)=H_{ij}(t) $$ the shortest H-path, and $$v_{ij,k^*}(t) \le v_{ij,k^2}(t) \le v_{ij,k^3}(t) \le \dots \le v_{ij,k^r}(t)$$ the $$r^{th}$$ shortest H-path. Then, for all $$k^* \in {\mathscr {K}}^{[m,n]}$$ and $$\beta ^*$$ fixed integer number of alternative stations we can compute the maximum percentage $$\alpha _{ij}^{\beta ^*}(k^*(t))$$ of the travel time $$H_{ij}(t)$$, such that $$| h \in {\mathscr {K}}^{[m,n]} : \frac{v_{ij,h}(t) - H_{ij}(t)}{H_{ij}(t)}<\alpha ^{\beta ^*}_{ij}(k^*(t))| < \beta ^*$$. Indicating $${\mathscr {P}}(k^*(t))$$ the number of shortest H-paths that change in $$k^*$$ at time *t*, we define $$\beta ^*$$*-dilemma factor* as

7 $$\begin{aligned} \alpha ^{\beta ^*}(k^*(t)) = \left( 1/{\mathscr {P}}(k^*(t)) \right) \sum _{i\ne j}^{{\mathscr {N}}} \alpha _{ij}^{\beta ^*}(k^*(t)), \end{aligned}$$

In a dual point of view, we define $\alpha ^*$*-dilemma factor for the station* $k^*$ the average number of alternative stations that implies a percentage $+\alpha ^* \%$ of travel time more than the shortest H-paths that pass through $k^*$. In formula:

8 $$\begin{aligned} \beta ^{\alpha ^*}(k^*(t)) = \left( 1/{\mathscr {P}}(k^*(t)) \right) \sum ^{{\mathscr {N}}}_{i\ne j}|R^{\alpha ^*}_{ij}(k^*(t))| \end{aligned}$$

where $R^{\alpha ^*}_{ij}(k^*(t))$ is the set of the alternative stations which their H-path from *i* to *j* is not longer than the $\alpha ^*\%$ of the shortest H-path at time *t*. The average of all the $\beta ^{\alpha ^*}(k^*(t))$ is the $\beta ^*$*-dilemma factor of the duplex* ${\mathscr {G}}^{[m,n]}$ at time *t*, that is $\beta _{[m,n]}^{\alpha ^*}(t) = \frac{1}{K^{[m,n]}}\sum _{k \in {\mathscr {K}}^{[m,n]}}\beta ^{\alpha ^*}(k^*(t))$. These two factors can be used in urban management to move closer to a system optimal traffic assignment by fixing the minimum number of alternatives or the maximum flexibility of users; in other words, to compute the price of anarchy in multimodal traffic dynamical systems. Moreover, we revealed a relation between these two factors that maintains linear behaviour for small values of $\beta ^*$ and $\alpha ^*$ at every level of congestion. As explained in detail in the following section, the angular coefficient $\Gamma (\alpha ^*,\beta ^*)$ between the two of them evaluates a general property of the whole transportation network in sense of adequate offer of multimodal path alternatives during severe traffic condition with limited users flexibility. For this reason, we called $\Gamma$ the *Network Congestion Elasticity*.

### Weighted dynamical efficiency

Until now, the definition of the dynamical efficiency has been based on an all-couples shortest H-paths algorithm, that corresponds to one and only one trip between all possible couples of nodes in the multilayer transportation network. If we want to quantify a user-oriented and not only network-oriented dynamical efficiency, we need an Origin-Destination (OD) matrix $Q^{[m,n]}(t)$, whose coordinates $q^{[m,n]}_{ij}(t)$ are proportional to the recorded or estimated traffic between locations *i* and *j* at time period indicate by *t* for each transportation mode ($m=n$) or coupled modes ($m\ne n$). In tha case where only the origin-destination information is know and not the mode choice, then we can assign $q^{[m,n]}_{ij}(t)= q_{ij}(t)$ for each mode $m,n \in [1,\dots , M]$. We will denote the weighted quantities for the dynamical efficiency with a tilde over them. For a location *i*, the *weighted node dynamical efficiency* at time *t* will be

9 $$\begin{aligned} {\tilde{e}}^{[m,n]}_i(t) = \frac{1}{|Q(t)|}\sum _{p \in {\mathscr {N}}^{[m,n]}\setminus \{i\}}{q^{[m,n]}_{ip}(t)\frac{\tau _{ip}^{[{\mathscr {M}}]}}{H^{[m,n]}_{ip}(t)}}, \end{aligned}$$

where $Q^{[m,n]}(t) = \sum _{p} \sum _{i}q^{[m,n]}_{ip}(t)$. For the link $\ell _{(i,j)}$, ${\tilde{E}}_{\ell _{(i,j)}}(t) = ({\tilde{e}}_i(t) + {\tilde{e}}_j(t))/2$. This measure gives us an idea of how many users experience a certain efficiency and at the same time reveals how the link betweenness changes according to the congestion and the traffic pattern. As we will describe later, ignoring these demand patterns can create counter-intuitive results. Nagurney et al.^33^ also introduced a similar measure of static efficiency for single layer networks by considering the flows of links. These flows were computed based on traffic assignment models of user equilibrium and not based on direct travel time measurements.

## A large-scale urban network application with real data

In this section, we show the results of the application of the multilayer dynamical efficiency definition in the case of the central part of Shenzhen, a large and important metropolis of China in Guangdong province where we have an extensive dataset of taxis GPS that gives us an accurate estimation of links speeds for the road network with a frequency of 5 minutes. The road network that we consider is composed of 2013 links and 1858 nodes and the metro network with 11 lines, 75 stations and 163 links. We note that many of the nodes in the road network are intermediate nodes and not all of them represent an intersection. Moreover, we extracted from OpenStreetMap detailed information of the metro system of the studied area about speed, frequency, location, distances (more details in SI, Figs. S1, S8, S9). For the sake of simplicity, we located every metro station to the closest node of the road network. We also took into account the walking time needed to go from a node to the closest metro station and added the average waiting time to the travel time through underground metro.

In order to show the potential of the dynamical efficiency in a multimodal transportation system, we also simulated how a distributed bike-sharing system with an arbitrary fixed number of stations (200) would improve the multi-layered transportation system of the city of Shenzhen in terms of absolute values and homogeneity of the level of service. For the details of this application and the results, please refer to Figs. S11 and S12 in SI.

**Figure 2:**
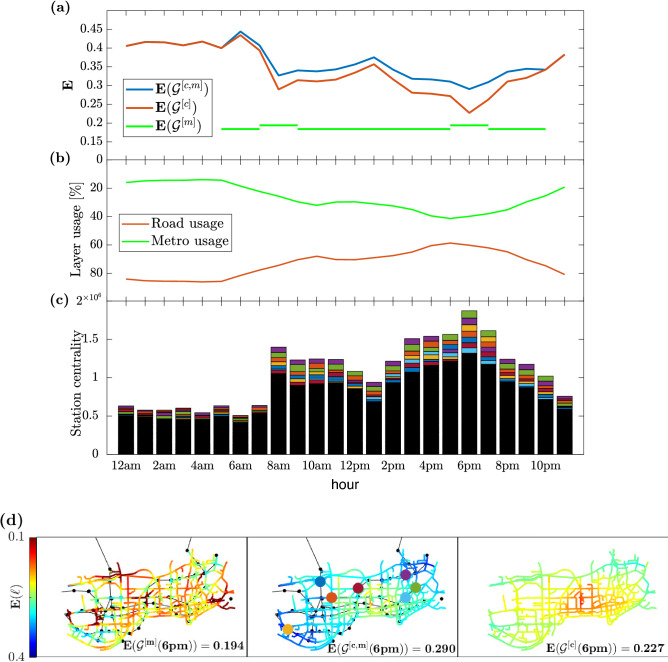
Temporal and spatial efficiency of an urban multimodal transportation system. (**a**) Temporal variation of the network dynamical efficiency for the city of Shenzhen (China) during a typical day (7th September 2011). Reported are the efficiency of the single car layer (${\mathscr {G}}^{[\mathrm{c}]}$) (red), the efficiency of the single metro layer (${\mathscr {G}}^{[\mathrm{m}]}$), which also considers walking and waiting times, and the peak and off peak frequency of the trains (green), and the efficiency of the combined multilayer system ${\mathscr {G}}^{[\mathrm{c,m}]}$, with the Eq. (4) of park and ride system (blue). (**b**) The average usage of each layer, namely the percentage of all travel time spent on car or metro, in the case of the combined multilayer system. (**c**) Sum over all intermodal junctions of their *station centrality*, that is the number of mode changes between road and metro networks of all H-paths passing by the station. (**d**) Spatial map of link efficiency for the three networks considered in panel (**a**). Blue colors mean higher efficiency, while red indicates lower efficiency with respect to the network free-flow condition. The seven most used stations (those with the largest station centrality) at 6pm have been highlighted with the same colors as in panel (**c**).

In panel (a) of Fig. 2, we plot the average road dynamical efficiency for vehicular traffic of the network during a whole day, specifically the 7th September 2011, in Shenzhen. As free flow condition, we set at 30 [mph] the maximum speed of all the links (being 29.81 [mph] the maximum link speed measured in our dataset). For the definition of dynamical efficiency, we compare the travel time in free flow condition with the effective travel time calculated with the available speed data. The results show the presence of two distinct peak hours (PH): one at 8 a.m. and the other, more severe, around 6 p.m. The average usage of each layer during the day is reported in panel (b) of Fig. 2. Here, for each hour, the average percentage of each shortest H-path belonging to road or to metro layer has been calculated. While in the histogram below (panel (c)), the amount of intermodal changes (i.e. the sum of all station centralities) during the day has been reported. We notice how road congestion increases the usage of public transportation. Panel (*d*) of Fig. 2 illustrates the coloured map of Shenzhen, based on the corresponding efficiency calculated for 3 different networks at evening peak hour: on the left, it represented the metro network efficiency, on the right the road efficiency and in the center the multilayer network of the integrated system of cars and metro. This panel clearly visualizes the increment in terms of service that each transportation layer brings to urban mobility, especially during the most congested hour. We want to remark that for the metro network the travel time from a location *i* to a destination *j* has been considered as the composite trip of walk and metro plus the waiting time at departing station and, whenever it is needed, for the metro line transfers (this is from the point of view of a traveler that uses only public transportation). For this reason, around the metro stations (black points) the efficiency is much higher than in the zones far from any public service.

**Figure 3:**
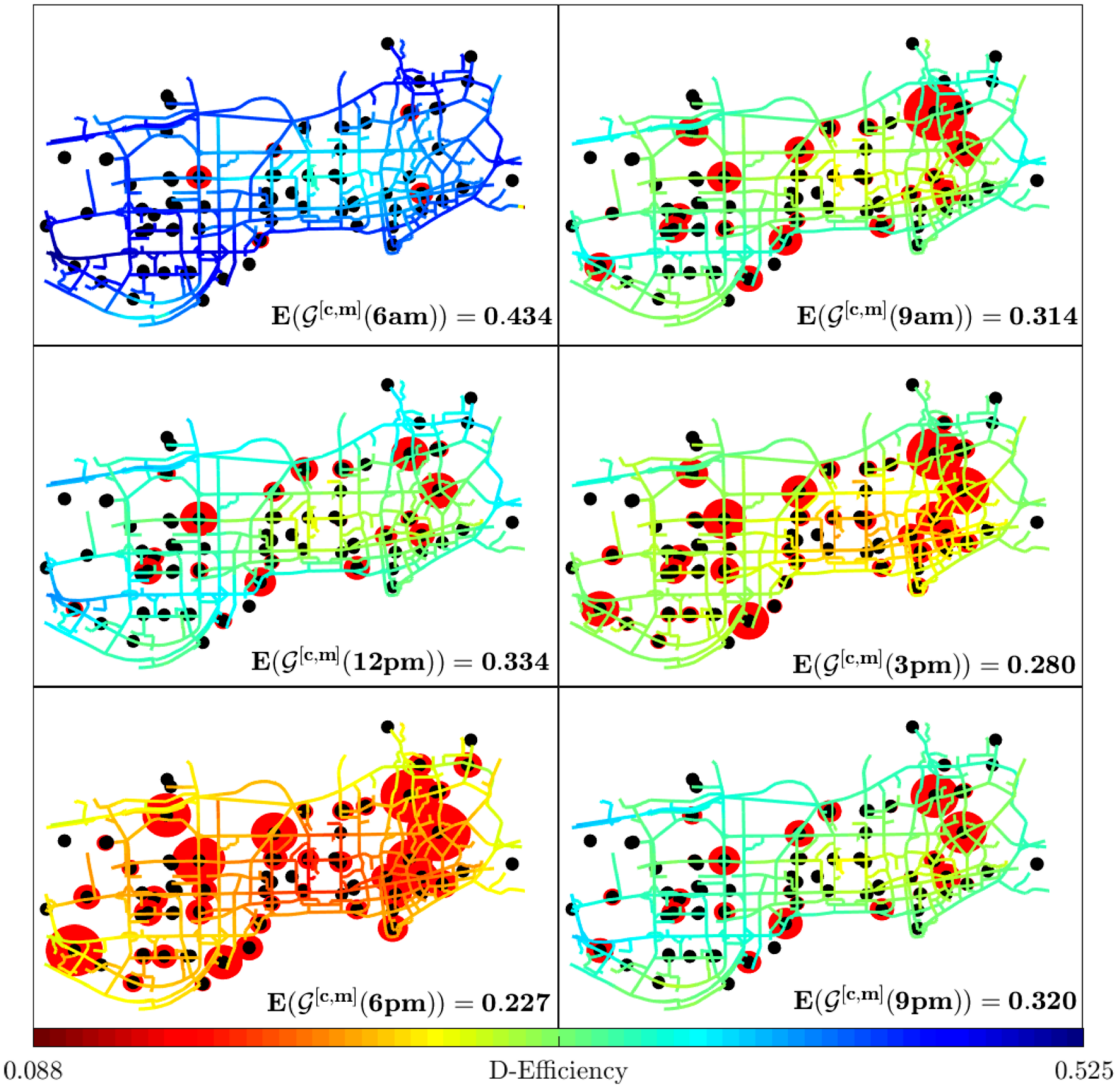
Daily variations of road efficiency and station centrality. The spatial patterns of road efficiency are shown at six different times of the day (from 6 a.m. to 9 p.m., every 3 h) for the city of Shenzhen (same day as in Fig. 2.) The color on each link stands for the efficiency of the corresponding road, from a minimum value of $E = 0.088$ in dark red, to a maximum value of $E= 0.525$ in dark blue. The center-east and the south-west parts of the network are those experiencing the most severe congestion, especially at rush hours. In the same map, the size of the red circles around the intermodal stations (black points) are proportional to the values of station centrality as defined in Eq. (6). We notice that the nodes with the highest station centrality are not inside, but around the congested areas of the city.

In Fig. 3 the distribution over the links of the dynamical efficiency values of the road layer is shown and, thanks to their geometrical smoothness, it is possible to follow the spreading of congestion over the network and visualize the perimeter of the congested zones. This represents one of the main differences with the link speeds map where there is no clear spatial value progression within adjacent links (SI, Figs. S5, S6).

**Figure 4:**
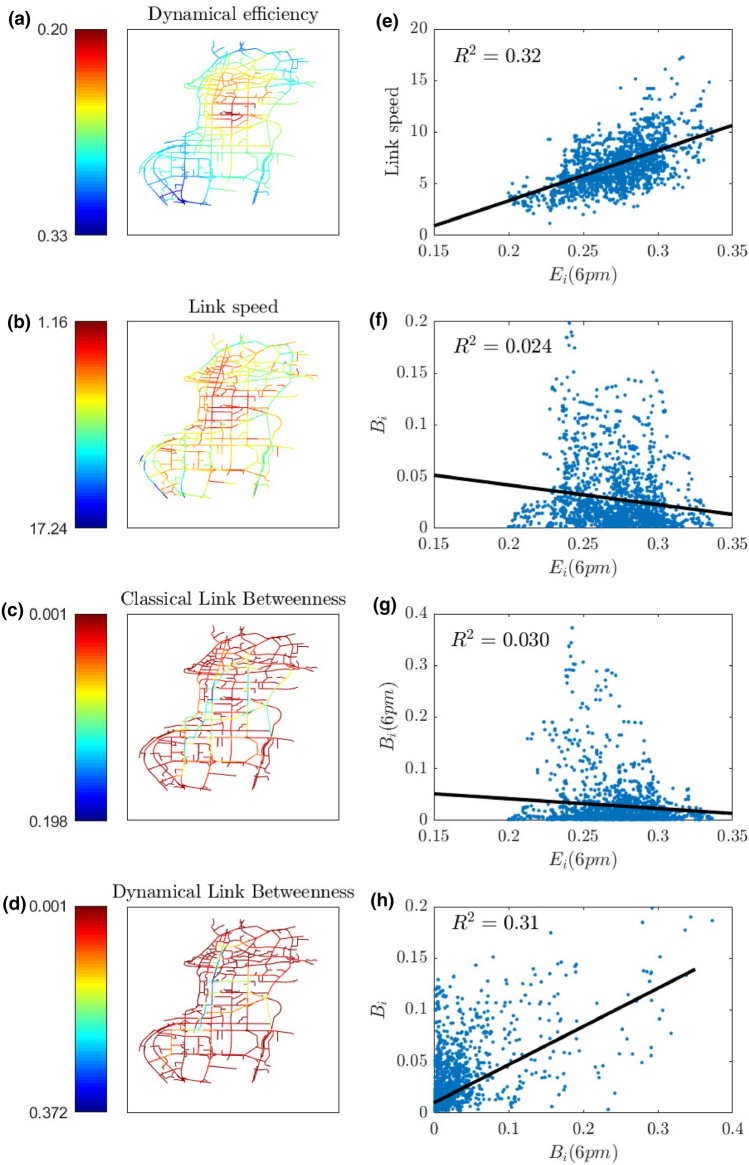
Comparison of the dynamical efficiency to betweenness and link speed. Panels (**a**)–(**d**) show the spatial distribution of the link values at 6pm for: dynamical efficiency, link speed, betweenness centrality and dynamical betweenness respectively. The scatter plots in panels (**e**) through (**g**) show the correlations between the dynamical efficiency and the other measures, while that in (**h**) refers to the correlation between classical and dynamical betweenness. Each blue point represents a link, while the black line is the linear regression of the plotted data. In each panel the value of the $R^2$ of the corresponding linear regression is also reported.

For a single layer of the network (road layer), we compare in Fig. 4, the spatial distribution of dynamical efficiency (Fig. 4a) to the distributions of link speed (Fig. 4b) and betweenness (without (Fig. 4c) and with (Fig. 4d) traffic). For panels (a), (b) and (d) we have considered the link travel times during the evening peak hour (6 p.m.). In panels (e)–(g) we report the scatter plots between the dynamical efficiency and the other measures. Each point refers to a link of the network. With respect to spatial distribution of link speed, the values of link dynamical efficiency are distributed in such a way that the most congested zones are clearly identified. Moreover, the betweenness centrality (Fig. 4c) does not appropriately describe the mobility characteristics of the network and the most critical links, not even when link travel times are considered as the weights of the graph (Fig. 4d). One of the features of the dynamical efficiency that distinguishes it from classical network centralities is its capacity of describing, on one hand, the variation in time of the network elements’ efficiency (links, stations) and, on the other hand, the evaluation of each link with respect to the whole network (distinguishable and smoothed congestion pattern).

We compute the shortest H-paths between all pairs of nodes considering the private car layer and the metro system. Every time a shortest H-path of type (*I*) or (*II*) passes through a station *k*, the value of the centrality of *k* (and *h* for *type-II*) increases. The size of the red spots in Fig. 3 is proportional to the station centrality at different times. As the congestion grows in the central area the centrality of the stations becomes more important. This measure, as already mentioned, can be fundamental to estimate the best station capacity and at the same time, in a park and ride system the size of the parking spots or their relative cost based on the time period in an optimized pricing policy. In this figure, we show how much this measure is sensitive to the congestion and how it recognizes the best location to change between car and metro. This is because in severe traffic condition the presence of public transportation alternatives is more suitable, especially for the aim to protect the central zones of a city from unmanageable traffic jams. Moreover, the station centrality information and the dilemma factor defined in Eqs. (7) and (8) can be used to regulate the distribution of users in a network and avoid unexpected accumulation and, consequentially, congestion.

**Figure 5:**
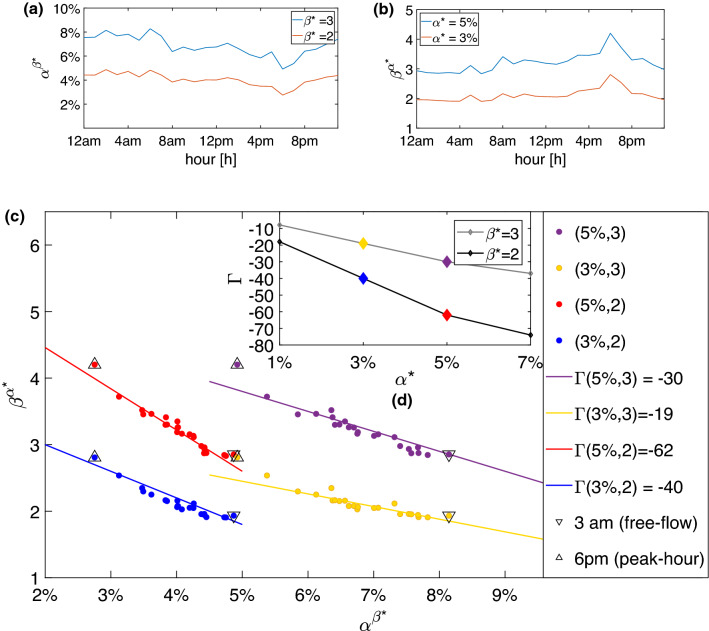
The $\alpha$ and $\beta$ dilemma factors and their correlations. Daily variations of the network dilemma factors (averages are taken over all the station nodes of the network) for two fixed values of $\beta ^*$ (**a**) and two different values of $\alpha ^*$ (**b**). Correlations between the two dilemma factors are studied by the scatter plot $(\alpha ^{\beta ^*},\beta ^{\alpha ^*})$ reported in panel (**c**). Each point here represents a different hour of the day, from midnight to 11 p.m. (peak-hour 6 p.m. and free-flow regime 3 a.m. are shown with up- and down-pointing triangles respectively), while the color indicates a different pair of values $\alpha ^*$, $\beta ^*)$. The linear relation between the two factors is highlighted by a linear fitting in the four studied cases. (**d**) The $\Gamma$ correlation coefficients obtained from the slope of the fitting in eight different cases corresponding to $\beta ^* = 2,3$ (grey and black) and four values of $\alpha ^* = 1,3,5,7 \%$. The colors of the four central diamonds correspond to the four cases reported in (**c**). $\Gamma$ quantifies the richness of the alternative paths that the transportation system offers to travellers if we allow paths with travel times $\alpha ^* \%$ longer than the shortest H-path.

Figure 5 represents the average of the $\beta ^*-$ (Fig. 5a) and $\alpha ^*-$ (Fig. 5b) dilemma factors during the day and the relation between these two measures. One can notice that both quantities change significantly only during the evening peak hour where the severity and the spatial distribution of traffic becomes critical. There exists a strong dependence between these two factors during the whole day and the congestion grows that we pointed out in Fig. 5c to be linear for small $\beta$. The slope of this dependence, that we indicate with $\Gamma (\alpha ^*,\beta ^*)$, stands for the influence that the congestion has on the dilemma factors. In particular, it is the variability, due to the congestion, of the $\alpha ^*$-dilemma factor (number of close stations) over the $\beta ^*$ factor (extra-time flexibility to have $\beta ^*$ paths options). So, we claim that $\Gamma$ evaluates the multilayer network not in terms of the efficiency (shortest travel times) but in terms of richness of close alternatives to the best H-path in all traffic conditions. The higher the $\Gamma$, the more the choice when congestion arrives with fixed users’ flexibility. Figure 5c shows that the relation between the two factors remains constant during the whole day, i.e. $\beta ^{\alpha ^*}(t) \propto \Gamma (\alpha ^*,\beta ^*) \alpha ^{\beta ^*}(t)$. We notice that the two factors are negatively correlated, $\Gamma (\alpha ^*,\beta ^*) <0$. In fact, when $\alpha ^{\beta ^*}$ reaches its minimum value (at 6 p.m.), $\beta ^{\alpha ^*}$ has its maximum peak. For example, $\alpha ^{\beta ^*=3}$ goes from $8\%$ of extra travel time at free flow condition to $5\%$ at peak hour (6 p.m.). This means that a user that in free flow condition needs to spend in average $8\%$ extra of its travel time to have at least 3 more alternative paths, during the peak hour it will spend only $5\%$ more than the its shortest H-path. On the other hand, Fig. 5b says that with a fixed travel time flexibility, for example $\alpha ^* = 5\%$, during peak hour the number of alternative paths goes from an average of 3 to an average of 5. Here, we reported the parameter $\Gamma (\alpha ^*,\beta ^*)$ only for the cases $\beta ^* =2,3$ and $\alpha = 3,5\%$. These are the most representative cases because they interpret the dependence on the extra travel time $\alpha ^*$ to have one or two more alternatives than the shortest H-path. Finally, the sub-panel (d) in Fig. 5d illustrates how the parameter $\Gamma$ scales for different values of factors $\alpha ^*= 1,3,5,7\%$ and $\beta ^*=2,3$.

**Figure 6:**
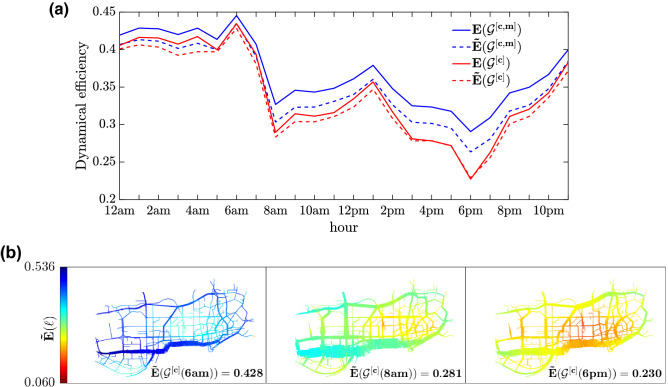
Daily variation and spatial distribution of weighted road efficiency. (**a**) The network dynamical efficiency *E* (continuous line) and its weighted version ${\tilde{E}}$ (dashed line) for the road network ${\mathscr {G}}^{[\mathrm{c}]}$ (red) and for the duplex network ${\mathscr {G}}^{[\mathrm{c,m}]}$ (blue) are plotted at different times of the day. (**b**) Spatial maps of road weighted efficiencies. The weighted dynamical efficiency of each link has been obtained by multiplying the dynamical efficiency of the link by its estimated flow. The colors correspond to the value of the weighted dynamical efficiency of the road system and the thickness of the road to their dynamical betweenness centrality considering the available OD information. We reported three different times of the day. At 6 a.m. the network experiences low demand and high efficiency (left). At 8am, the demand grows (notice the increase in link betweenness of roads in the southern part of city highway) but not enough to create a severe congestion (middle). Finally, at 6 p.m. the congestion forces travellers to find alternative paths to the highway.

By adding the information of daily demand between different zones of the city and calculating the usage of a road based on the estimated volume of traffic, we reported in Fig. 6a the comparison between the dynamical efficiency (*E*(*t*)) and the weighted dynamical efficiency (${\tilde{E}}(t)$) and reconstructed in Fig. 6b the map of weighted efficiency defined in Eq. (9). The origin-destination (OD) matrix that we used is based on more than 200k taxis trips par hour for a whole day with peaks of 13k at 9 a.m. and 6 p.m. The thickness of the roads represented in Fig. 6b is proportional to the amount of shortest H-paths passing through it and the color represents the corresponding link dynamical efficiency. We notice that in general $E(t) < {\tilde{E}}(t)$, this is because more travellers use the overloaded road and these count more in the average formula for ${\tilde{E}}$ than for *E*. The methods and the results of this OD estimation are illustrated in the dedicated section of SI (Figs. S8 and S9).

## Conclusion

A distinctive feature of urban transportation networks is the complex interplay among their physical structure (e.g. roads, public transport, bike-sharing stations), the traffic demand (e.g. origin-destination matrix) and the traffic condition (e.g. link speed data). While many works have focused on each of these aspects considered separately, we aimed instead at the definition of single measures for time-varying transportation networks, such as the dynamical efficiency proposed in this article, that can embrace all the aspects at once. This makes such measures particularly suited to practical applications in real world case studies.

One of the key points of this paper is the definition of the H-paths. These paths correspond to trips taking place over two layers of a transportation network, with either one or two intermodal changes. Examples of H-paths are park and ride schemes, or trips coupling public transportation systems (bus, metro, etc.) with the first and/or the last mile made by walk or bike. From the point of view of the computational cost, the evaluation of the all shortest H-paths has some advantages, as the all-shortest path algorithm needs to be applied to each layer individually and not to the whole multilayer network (see Fig. S3 in SI). In addition to this, H-paths allow to describe the majority of real urban trips and to define other important measures like the station centrality and the dilemma factor. The station centrality quantifies how convenient is to change in a station from a layer to another at each time *t*, and can be easily implemented in on-line mobile applications (as proposed, for example, in^48^), or used for parking spot management and pricing^31,49^, or for station capacity regulation and optimized traffic control strategies. The second quantity, the dilemma factor, measures the richness of equivalent transportation alternatives and can be used in traffic assignment management and control to quantify the feasibility of some path recommendation and route pricing strategies^50^. In particular, this measure quantifies the set of sub-optimal paths among travelers’ choice in a bounded rationality framework for traffic assignment models as in^51,52^. For each station, the dilemma factor is all about the conversion rate between the extra travel time $\alpha ^*$ with respect to the shortest H-path and the number $\beta ^*$ of alternatives. We noticed that this rate changes during the day because of the congestion but that, if we look at the entire network for small values of $\alpha ^*$ and $\beta ^*$, the relation between the increase in congestion and the rate change is linear (the so-called *network congestion elasticity* $\Gamma (\alpha ^*, \beta ^*)$). This means a more accurate prediction on users’ adhesion to traffic control strategies with a data-driven travel time flexibility $\alpha ^*$ and an estimation of the number of the trip alternatives $\beta ^*$ that the urban mobility, in its multi-level complexity, offers under congested conditions.

Another factor that might influence the dynamical efficiency and the network congestion elasticity is the introduction of a new transportation mean (e.g. bus system, bike-sharing) that changes not only the topology of the multilayer network but also the link travel times and their intermodal traffic dependence making the whole system more or less elastic to congestion stress. An example on the effect that the introduction of a bike-sharing system with fixed stations would have on the network dynamical efficiency is shown in Figs. S11–S12 of SI. We are confident that other insightful results can come from the comparative analysis of multilayer transportation systems of different cities.

The strength of the dynamical efficiency in single and multilayered networks is that it merges the network topology (betweenness, H-path, intermodal junctions) and the dynamics of traffic captured by high frequency link speed data (instantaneous travel time). These two combined factors differentiate our definition of *efficiency* from the static measures existing in the literature^7,17,33,38,42^ and from more recent works based on the annual average traffic estimation (e.g. Ref.^53^) that look at long-term transport planning applications and not at the daily traffic pattern changes. Another advantage is that the dynamical efficiency of a network can be weighted also based on the real or estimated origin-destination flows. We showed how the demand pattern may influence not only the global performance of the transportation systems but also the usage of roads and stations. Given an OD matrix *Q*(*t*) Eq. (9) returns the weighted dynamical efficiency of a given location *i* at time *t*. This opens new perspectives on the applications of traffic models that take into account not only the hour-by-hour link speed map but also the traffic flow among different zones of the city during the day (i.e. on-/off-peak hour, morning/evening, day/night, working days/weekend, bad/good weather conditions, etc.). It should also be highlighted that if trajectory data with high penetration rates is available, real paths of travellers will be known and the same metrics can be estimated without the need to run shortest-path algorithms. Moreover, the values of the dynamical efficiency are spatially smoothed through the network and this is useful to visualize the congested zones where and when to apply strategies that involve clustering algorithms and congestion perimeter delimitation.

Another aspect of the superiority of dynamical efficiency in real-world applications is its robustness with respect to sparse data errors and network percolation. We calculated the dynamical efficiency of the same Shenzhen’s network after performing 50 times a random percolation (of *P* links) per each $P = 40, 80 ,120 ,160, 200$. The results reported in Figs. S13–S17 of SI, show that, on the one hand, the average value of network dynamical efficiency decreases smoothly and proportionally with the number of percolated links and, on the other hand, that the spatial configuration of the dynamical efficiency pattern during the day can be highly influenced by the link removal. In other words, that the dynamical efficiency keeps regularity and robustness characteristics when we look at the network features (i.e. average value) while describing with immediate and clear feedback the local effects of congestion.

As shown in very recent works^4,5^, the congestion tends to propagate from some distributed seeds to adjacent links through diffusion-like process. Moreover, it has been observed that close links influence each others traffic state: that is, a link adjacent to a congested link has high probability to become congested later on. This suggests that it is worth investigating the relation between links with high dynamical efficiency and their probability to get congested. In this direction, the dynamical efficiency can be used to design a predictive model for link and station congestion. Finally, the user-oriented efficiency proposed in this study is a practical and powerful mean to calibrate and estimate the mobility in the city and to describe the path distribution needed to optimize mobility flows.

## Supplementary information

Supplementary Information.

## Data Availability

The data that support the findings of this study are available from the corresponding author upon request.

## References

[1] Mazloumian A, Geroliminis N, Helbing D (2010). The spatial variability of vehicle densities as determinant of urban network capacity. Philos. Trans. R. Soc. A Math. Phys. Eng. Sci..

[2] Saeedmanesh M, Geroliminis N (2017). Dynamic clustering and propagation of congestion in heterogeneously congested urban traffic networks. Transp. Res. Proc..

[3] Loder A, Ambühl L, Menendez M, Axhausen KW (2019). Understanding traffic capacity of urban networks. Sci. Rep..

[4] Bellocchi L, Geroliminis N (2020). Unraveling reaction–diffusion-like dynamics in urban congestion propagation: insights from a large-scale road network. Sci. Rep..

[5] Saberi M (2020). A simple contagion process describes spreading of traffic jams in urban networks. Nat. Commun..

[6] Gonzalez MC, Hidalgo CA, Barabasi A-L (2008). Understanding individual human mobility patterns. Nature.

[7] Crucitti, P., Latora, V. & Marchiori, M. Model for cascading failures in complex networks. *Phys. Rev. E* **69**, 045104 (2004). 10.1103/PhysRevE.69.045104 15169056

[8] Manfredi S, Di Tucci E, Latora V (2018). Mobility and congestion in dynamical multilayer networks with finite storage capacity. Phys. Rev. Lett..

[9] Ash J, Newth D (2007). Optimizing complex networks for resilience against cascading failure. Phys. A.

[10] Buldyrev SV, Parshani R, Paul G, Stanley HE, Havlin S (2010). Catastrophic cascade of failures in interdependent networks. Nature.

[11] Zeng G (2019). Switch between critical percolation modes in city traffic dynamics. Proc. Nat. Acad. Sci..

[12] Zhang, L. *et al.* Scale-free resilience of real traffic jams. *Proc. Natl. Acad. Sci.* **116**, 8673–8678. 10.1073/pnas.1814982116 (2019) 10.1073/pnas.1814982116 PMC6500150 30979803

[13] Li D (2015). Percolation transition in dynamical traffic network with evolving critical bottlenecks. Proc. Nat. Acad. Sci..

[14] Mishra S, Welch TF, Jha MK (2012). Performance indicators for public transit connectivity in multi-modal transportation networks. Transp. Res. Part A Policy Pract..

[15] Solé-Ribalta, A., Gómez, S. & Arenas, A. A model to identify urban traffic congestion hotspots in complex networks. *R. Soc. Open Sci.* **3**, 160098 (2016). 10.1098/rsos.160098 PMC5098960 27853535

[16] Viana MP, Strano E, Bordin P, Barthelemy M (2013). The simplicity of planar networks. Sci. Rep..

[17] Crucitti, P., Latora, V. & Porta, S. Centrality measures in spatial networks of urban streets. *Phys. Rev. E* **73**, 036125 (2006). 10.1103/PhysRevE.73.036125 16605616

[18] Scellato S, Fortuna L, Frasca M, Gómez-Gardenes J, Latora V (2010). Traffic optimization in transport networks based on local routing. The European Physical Journal B.

[19] Çolak S, Lima A, González MC (2016). Understanding congested travel in urban areas. Nat. Commun..

[20] Lima A, Stanojevic R, Papagiannaki D, Rodriguez P, González MC (2016). Understanding individual routing behaviour. J. R. Soc. Interface.

[21] Alonso-Mora J, Samaranayake S, Wallar A, Frazzoli E, Rus D (2017). On-demand high-capacity ride-sharing via dynamic trip-vehicle assignment. Proc. Nat. Acad. Sci..

[22] Calabrese, F., Di Lorenzo, G., Liu, L. & Ratti, C. Estimating origin-destination flows using opportunistically collected mobile phone location data from one million users in Boston metropolitan area (2011).

[23] Calabrese F, Diao M, Di Lorenzo G, Ferreira J, Ratti C (2013). Understanding individual mobility patterns from urban sensing data: A mobile phone trace example. Transp. Res. Part C Emerging Technol..

[24] Kurant, M. & Thiran, P. Extraction and analysis of traffic and topologies of transportation networks. *Phys. Rev. E* **74**, 036114. 10.1103/PhysRevE.74.036114 (2006). 10.1103/PhysRevE.74.036114 17025715

[25] Modesti P, Sciomachen A (1998). A utility measure for finding multiobjective shortest paths in urban multimodal transportation networks. Eur. J. Oper. Res..

[26] Krygsman S, Dijst M, Arentze T (2004). Multimodal public transport: An analysis of travel time elements and the interconnectivity ratio. Transp. Policy.

[27] Geroliminis N, Zheng N, Ampountolas K (2014). A three-dimensional macroscopic fundamental diagram for mixed bi-modal urban networks. Transp. Res. Part C Emerging Technol..

[28] Loder A, Ambühl L, Menendez M, Axhausen KW (2017). Empirics of multi-modal traffic networks-using the 3d macroscopic fundamental diagram. Transp. Res. Part C Emerging Technol..

[29] Geroliminis N, Daganzo CF (2008). Existence of urban-scale macroscopic fundamental diagrams: Some experimental findings. Transp. Res. Part B Methodol..

[30] Buisson C, Ladier C (2009). Exploring the impact of homogeneity of traffic measurements on the existence of macroscopic fundamental diagrams. Transp. Res. Rec..

[31] Zheng N, Geroliminis N (2016). Modeling and optimization of multimodal urban networks with limited parking and dynamic pricing. Transp. Res. Part B Methodol..

[32] Kouvelas A, Saeedmanesh M, Geroliminis N (2017). Enhancing model-based feedback perimeter control with data-driven online adaptive optimization. Transp. Res. Part B Methodol..

[33] Nagurney A, Qiang Q (2012). Fragile networks: identifying vulnerabilities and synergies in an uncertain age. Int. Trans. Oper. Res..

[34] Erman, J., Arlitt, M. & Mahanti, A. Traffic classification using clustering algorithms. in *Proceedings of the 2006 SIGCOMM workshop on Mining network data*, 281–286 (ACM, 2006).

[35] Sirmatel II, Geroliminis N (2017). Economic model predictive control of large-scale urban road networks via perimeter control and regional route guidance. IEEE Trans. Intell. Transp. Syst..

[36] Aalipour, A., Kebriaei, H. & Ramezani, M. Analytical optimal solution of perimeter traffic flow control based on MFD dynamics: A pontryagin’s maximum principle approach. *IEEE Trans. Intell. Transp. Syst.* (2018).

[37] Freeman, L. C. A set of measures of centrality based on betweenness. *Sociometry* (1977).

[38] Barthelemy M (2004). Betweenness centrality in large complex networks. Eur. Phys. J. B.

[39] Barthélemy M (2014). Spatial Networks.

[40] Kirkley A, Barbosa H, Barthelemy M, Ghoshal G (2018). From the betweenness centrality in street networks to structural invariants in random planar graphs. Nat. Commun..

[41] Kivelä M (2014). Multilayer networks. J. Complex Netw..

[42] Battiston, F., Nicosia, V. & Latora, V. Structural measures for multiplex networks. *Phys. Rev. E* **89**, 032804 (2014). 10.1103/PhysRevE.89.032804 24730896

[43] Boccaletti S (2014). The structure and dynamics of multilayer networks. Phys. Rep..

[44] De Domenico M, Solé-Ribalta A, Omodei E, Gómez S, Arenas A (2015). Ranking in interconnected multilayer networks reveals versatile nodes. Nat. Commun..

[45] De Domenico M, Granell C, Porter MA, Arenas A (2016). The physics of spreading processes in multilayer networks. Nat. Phys..

[46] Owen A, Levinson DM (2015). Modeling the commute mode share of transit using continuous accessibility to jobs. Transp. Res. Part A Policy Pract..

[47] Youn, H., Gastner, M. T. & Jeong, H. Price of anarchy in transportation networks: efficiency and optimality control. *Phys. Rev. Lett.* **101**, 128701 (2008). 10.1103/PhysRevLett.101.128701 18851419

[48] Minea, M. G., Bădescu, I. G. & Dumitrescu, S. D. Efficiency of multimodal real-time travel and traffic information services employing mobile communications. In *2011 10th International Conference on Telecommunication in Modern Satellite Cable and Broadcasting Services (TELSIKS)*, vol. 2, 765–768 (IEEE, 2011).

[49] Liu W, Yang H, Yin Y (2015). Efficiency of a highway use reservation system for morning commute. Transp. Res. Part C Emerging Technol..

[50] Liu W, Yang H, Yin Y, Zhang F (2014). A novel permit scheme for managing parking competition and bottleneck congestion. Transp. Res. Part C Emerging Technol..

[51] Ameli, M., Lebacque, J.-P. & Leclercq, L. Improving traffic network performance with road banning strategy: A simulation approach comparing user equilibrium and system optimum. *Simul. Model. Pract. Theory* **99**, 101995 (2020).

[52] Kahneman, D. & Tversky, A. Prospect theory: An analysis of decision under risk. In *Handbook of the Fundamentals of Financial Decision Making: Part I*, 99–127 (World Scientific, 2013).

[53] Ganin, A. A. *et al.* Resilience and efficiency in transportation networks. *Sci. Adv.* **3**, e1701079 (2017). 10.1126/sciadv.1701079 PMC5744464 29291243

