# Supplementary material for: Dynamical efficiency for multimodal time-varying transportation networks
Source: Sci Rep. 2021 Nov 29;11:23065. doi: 10.1038/s41598-021-02418-5 (PMC8630039; doi:10.1038/s41598-021-02418-5)
Supplement: Supplementary file 1 — Supplementary Information. [file 41598_2021_2418_MOESM1_ESM.pdf]

Supplementary Information for

Dynamical efficiency for multimodal time-varying transportation networks

Bellocchi Leonardo, Latora Vito and Geroliminis Nikolas

Corresponding author: Nikolas Geroliminis
E-mail: nikolas.geroliminis@epfl.ch

This PDF file includes:

Supplementary text
Figs. S1 to S17

Supporting Information Text

Shenzhen multilayered transportation network. In order to build the transportation network of our case study in Shenzhen, we merged the information from OpenStreetMap with the details of the local metro line. Then, we connected each metro station to the closest node/intersection of the road network. The estimation of the node-to-node travel time comes from an extensive dataset of taxis GPS data that, after processing them with a map matching algorithm, gave us an estimation of links speeds for the road networks every 5 minutes. The road network that we consider is composed of 2013 links and 1858 nodes and the metro network with 8 lines, 75 stations and 163 links (Figure S1). We note that many of the nodes in the road network are intermediate nodes and not all of them represent an intersection. Moreover, we extracted from detailed information of the metro system speed, frequency of lines, location of stations and distances between stations. There exist 2 different frequencies of the metro that determine a different waiting time at the metro station, in particular, at on-peak ($[6am - 8am], [4pm - 6pm]$) the average time headway is 3 minutes and for the rest of the day (off-peak) is 6min. We also took into account the walking time needed (with 3.1 mph walking speed) to go from a node to the closest metro station and added the average waiting time to the travel time via public transport .

H-shortest path algorithm. In the main text, we declared our reasons to choose H-shortest path instead of classical shortest path in a multilayered network. Here, we explain the algorithm for both *type-I* and *type-II* that decreases dramatically the computational effort required. In Fig. S3, we report the computational time for the all shortest path algorithm in multilayer network compared to the all H-shortest path algorithm defined here. While the classical path searching requires a computational time that grows according to a power law with the number of layers, with our algorithm has a linear growth.

We consider H-paths as paths between two nodes that belong only to one or two layers that, for the sake of notation, we indicate here as $\mathcal{G}^{[1]}$ and $\mathcal{G}^{[2]}$. We notice that a layer can be the result of the aggregation of two or more single layers where the distance between two nodes of the aggregated network is the fastest possible using the best combination of duplex in the ensemble of networks. As an example, the time to go from a node O to a node D can be, in the aggregated network, the sum of the walking time to a metro station, the travel time spent in the metro and last mile by bike-sharing service, if this was the best combination using this three modes of transportation at that path between two location is not feasible in one layer, we set their relative distance equal to infinity. Once we have the structure of layer $\mathcal{G}^{[1]}$ and $\mathcal{G}^{[2]}$ and their corresponding link travel times $\mathcal{W}(t)$, it is possible to consider the matrix $N \times N$, $D^{[1]}(t)$ and $D^{[2]}(t)$, of the relative node-to-node distances at time t. In order to compute the H-shortest path of type I between a node i and a node j, it is sufficient to sum element by element the row $\mathbf{i}^{[1]}(t)$ of matrix $D^{[1]}(t)$ with the column $\mathbf{j}^{[2]}(t)$ of matrix $D^{[2]}(t)$. We obtain a vector $\mathbf{s}_{ij}^{[1,2]}(t) = (\mathbf{i}^{[1]}(t))^T + \mathbf{j}^{[2]}(t)$, where each coordinate k represents the travel time from i to j passing from layer $\mathcal{G}^{[1]}$ to layer $\mathcal{G}^{[2]}$ through station k at time t. The minimum value of vector $\mathbf{s}_{ij}^{[1,2]}(t)$ is the travel time of the H-shortest path and $k^* = argmin(\mathbf{s}_{ij}^{[1,2]}(t))$ the most convenient station where, eventually, to change (if $k^* \neq i, j$). Moreover, if we consider also the alternative path with a travel time close to the minimum we can obtain the dilemma factor as explained in the main text.

With this algorithm the computational effort decreases dramatically. In fact, it is sufficient to compute the all shortest path algorithm in every single layer and then, for each couple of Origin-Destination (i, j) to sum 2 vectors of dimension N and find the minimum value. The algorithm is illustrated schematically in Fig. S2.

For H-shortest path of type$-II$, that is paths that start from a layer $\mathcal{G}^{[1]}$, changes in k to a second layer $\mathcal{G}^{[2]}$ and then, again, in h to return to $\mathcal{G}^{[1]}$. The algorithm, in this second case, becomes as follows. Let us assume, without lose generality, that $\mathcal{N}^{[1]} \subseteq \mathcal{N}^{[1]}$ and $K = |\mathcal{K}^{[1,2]}| = |\mathcal{N}^{[2]} \cap \mathcal{N}^{[1]}|$. We can always consider the fat matrix $D^{[2,1]} \in \Re^{K \times N^{[1]}}$ of the time distance between node $i \in \mathcal{N}^{[1]}$ and node $j \in \mathcal{N}^{[2]}$. In this case, adding to each column of the matrix $D^{[2]}(t) \in \Re^{K \times K}$ the column $\mathbf{i}^{[2,1]}(t)$ of matrix $D^{[2,1]}(t)$ and to each row the row $\mathbf{j}^{[1,2]}(t)$ of the tall matrix $D^{[1,2]}(t) = (D^{[2,1]}(t))^T$, we obtain a $K \times K$ matrix $S_{ij}(t)$, whose coordinate (k, h) is the travel time to go from node $i^{[1]}$ to $j^{[1]}$ using line (h, k) of layer $\mathcal{G}^{[2]}$. The minimum value of matrix $S_{ij}(t)$ will be the H-shortest part of type$-II$ and the position (k^*, h^*) the best choice to effectuate the layer changes between $\mathcal{G}^{[1]}$ and $\mathcal{G}^{[2]}$.

Efficiency VS Speed. We want, here, to show the difference at local level, between dynamical efficiency and speed data. The network averages of these two measures, reported in Fig. S4, appear very similar but scaled in the case of efficiency with the maximum speed of 30 mph. It is at the link level where we appreciate the main differences. If we compare Fig. S5 and Fig. S6 we notice that the dynamical efficiency delimits the congested zones and it is smoothly distributed in the map. On the other side, the speed map shows confusing and heterogeneous distribution of the links' values.

Dilemma factor. In the main paper, we showed the equation and the general results for the α and β dilemma factors, and also the relation between the two of them. Here, for completeness, we want to show how, at local level, the α dilemma factor (and consequentially the β for the relation revealed in the main paper) is spatially distributed during the day among the intermodal junctions. In our case, the stations are the interchanges between road and metro system and $\alpha = 5\%$. In Fig. S7, the size of the red spots is proportional to the number of alternative stations with an extra travel time less than the 5% than the H-shortest path that passes through the spotted station. In this sense the dilemma factor become a measure of substitutability of each stations. As we suggest in the conclusion of our work, we believe that it is interesting and useful to study the feasibility of routing strategy, pricing and disruption cascade effects.

Daily dynamical efficiency variation. For each link $i \in N$, we define daily dynamical efficiency variation $\{\Delta E_i\}_{i \in N}$, as $\Delta E_i = \max_{t \in T} E(l(t)) - \min_{t \in T} E(l(t))$. In order to show how our measure of link dynamical efficiency differs for classical

network measures, we reported in the bottom panel of Fig S10b the distribution of $\{\Delta E_i\}_{i \in N}$. In a static scenario, where congestion does not influence the traffic performance of a urban network, the value of $\Delta E_i = 0$, for each $i \in N$. In order to show the difference between efficiency and the link speed values and the betweenness centrality, often used in transportation networks to evaluate the traffic performance, we plot in Fig. 4 of the paper the coloured map of Shenzhen and the correlation plot between these link measures. We notice that there are substantial differences in centrality when we consider the network with link speed value, in particular during the peak hour (6pm). In the scatter plots on the right of Fig. 4 of the paper we observe a weak ($R^2 = 0.32$) correlation between Dynamical efficiency and link speed values and no correlation ($R^2 \leq 0.03$) with the betweenness centrality with (f)) or without (g)) considering link speed data.

Bike layer

In this section, we illustrate an extension of the work presented in the main paper. The main purpose of this part is to give an illustrative example of both the flexibility of the computation of the dynamical efficiency in multi-layered networks and also one application of this measure to design optimized multimodal transportation systems. In particular, we add a new layer to the multiplex considered before, composed of walk, metro and private car layer. This new layer would represent an hypothetical bike-sharing system with 200 fixed stations distributed in the road network of Shenzhen. The heuristic method that we followed to locate the bike stations (Fig. S11a) is based on three principles: (A) optimize and homogenize the spatial link speed distribution of the Public Transportation network (Walk + metro) by placing the bike stations in the zones less served by PT; (B) locate the bike station in correspondence to metro stations for a practical intermodal change; (C) The bike stations should be at least 5 min biking (10km/h) far from each other. So, starting from the metro stations, we located a bike station in the nodes with the lowest dynamical efficiency, always respecting principle (C), until we reached the fixed (arbitrary) limit of 200 stations. The results are shown in Fig. S12. In particular, in panel (a) on the right it is shown the increment in terms of the average of percentage of private car trip of the all shortest H-path algorithm between the multilayer network composed by road/walk/metro (in red) and the same with the addition of the bike layer (in blue). On the right in colored green area, we highlight the reduction of car trip that we expected with the addition of a bike-sharing system such as those one explained before. Panel (b) of the same figure illustrates the impact the an additional bike-sharing layer (on the left) would have in the distribution of dynamical efficiency of the aggregated public transportation network walk/metro (on the right). We notice a general increase of dynamical efficiency with a less inequity among the links of the network.

Robustness

In order to test the robustness of dynamical efficiency, we used a link percolation process on the complete Shenzhen's network. Being $p = 40, 80, 120, 200$ the number of link randomly removed from the network (50 instances per each p), we measured: A) the network dynamical efficiency $E^{[c]}_{\setminus p}(t)$, (continuous line in Fig S12a-b); B) the standard deviation among all the 50 instances (dashed line in Fig S12a-b); D) the max and min value $\max_t E^{[c]}_{\setminus p}(t)$, $\min_t E^{[c]}_{\setminus p}(t)$ (Fig. S13); C) the dynamical efficiency pattern at time $t =$ 6am, 9am, 12pm, 3pm, 6pm, 9pm. (Fig S14-16).

Materials

Origin-Destination information. From the same dataset of taxis GPS used to estimate the link speeds, we extracted the origin and the destination location of each trip passing through the studied zone of Shenzhen. We considered not only the trips the have origin and destination in that zone but also the trip that start and/or end in the external part, but pass through it. The number of trip per hour changes as reported in Fig. S8a. The length of the majority of the trips is around 2km and the distribution is plotted in Fig. S8b. We assign in this case, the origin to the zone of the first point in the map and destination the last zone containing a GPS signal. In this way, we were able to collect around 220k taxis trips for the 7th of September and divided into the microzones as showed in Fig. S9a. The zone-to-zone traffic of the aggregated data is visualized in the circular graph S9b where the colour and the thickness are proportional to the demand between two zones.

Fig. S1. The road map of Shenzhen downtown with the 9 central metro lines in different colors. The road network that we consider is composed of 2013 links and 1858 nodes and the metro network with 8 lines, 75 stations and 163 links.

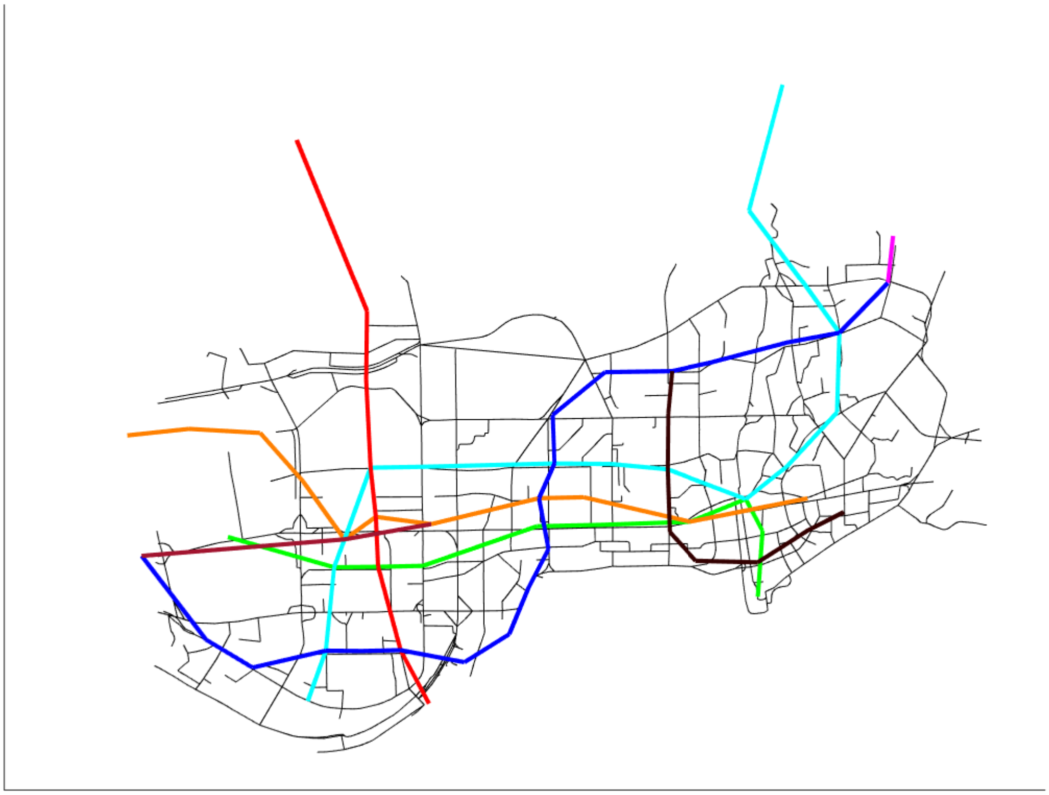

Fig. S2. Schematic representation of the algorithm to compute the H-shortest path type I and II between two layers.

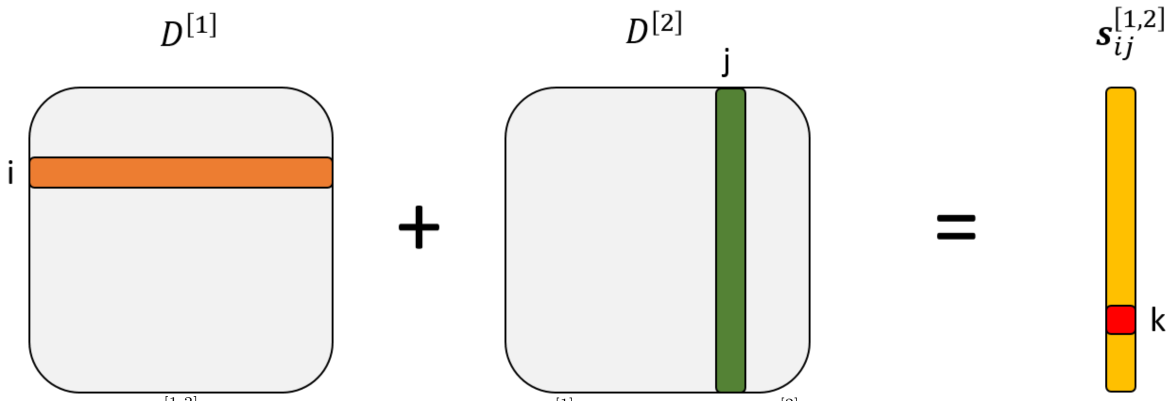

(a) *Type I.* The vector $\mathbf{s}_{ij}^{[1,2]}$, is the sum of row i of time distance matrix $D^{[1]}$ and column j of matrix $D^{[2]}$. The minimum value of **s**, located in k, is the travel time of the H-shortest path required between i and j, from layer $[1]$ to layer $[2]$. All the other entries h of the vector **s** are the H-paths between i and j changing in h. If $k == i$ the H-shortest path belong all to layer $[2]$, and viceversa if $k == j$, to layer $[1]$.

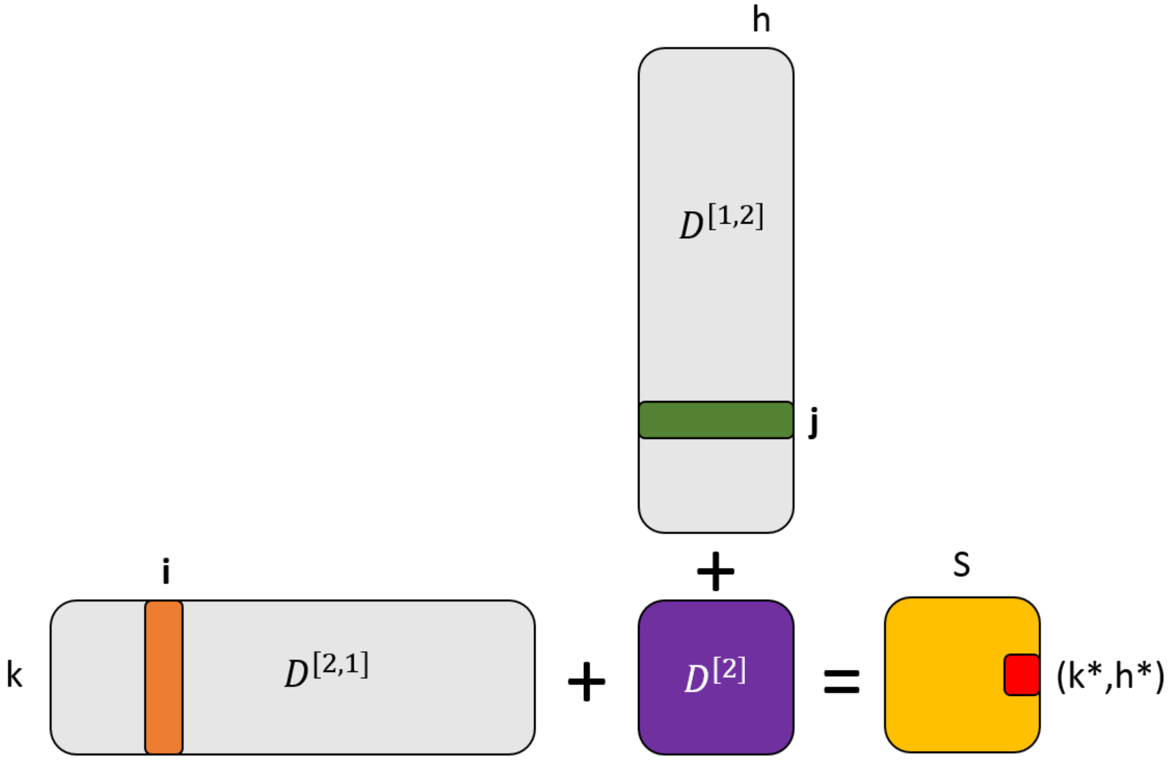

(b) *Type II.* The travel time of the H-shortest path of type II between two layers will be the minimum value (k^*, h^*) of the $K \times K$ matrix S, where $K \in N^{[1]} \bigcap N^{[2]}$ is the number of stations in layer $[2]$. Matrix S is composed by adding to each column of matrix $D^{[2]}$ the column **i** of matrix $D^{[2,1]}$ of the travel time between stations of layer $[2]$ and nodes of layer $[1]$ and to each of its rows the row **j** of matrix $D^{[1,2]}$ (from nodes $N^{[1]}$ to stations K).

Fig. S3. Computational time for classical all-shortest path algorithm in multilayered network and using H-path defined in the paper. To demonstrate the superiority of our algorithm, in computational sense, we tested 10 times the two different algorithms in a multilayered network with 1 -2 -3 -4 and 5 layers of similar road networks of 2013 links and 1858 nodes (the same used in the paper). Between two layers, we randomly chose at each iteration half of the nodes in one layer and connected them with their corresponding images in the other layer. For each case, the corresponding boxplot of computational time [sec] is reported. The classical all-shortest path algorithm takes a time that goes with a power law ($f(x) = 0.10x^{2.32}$) respect to the number of layers, while the H-path goes linearly with it. For example, for the case of 5 layers the H-path algorithm goes around x8 faster that the classical all shortest path search.

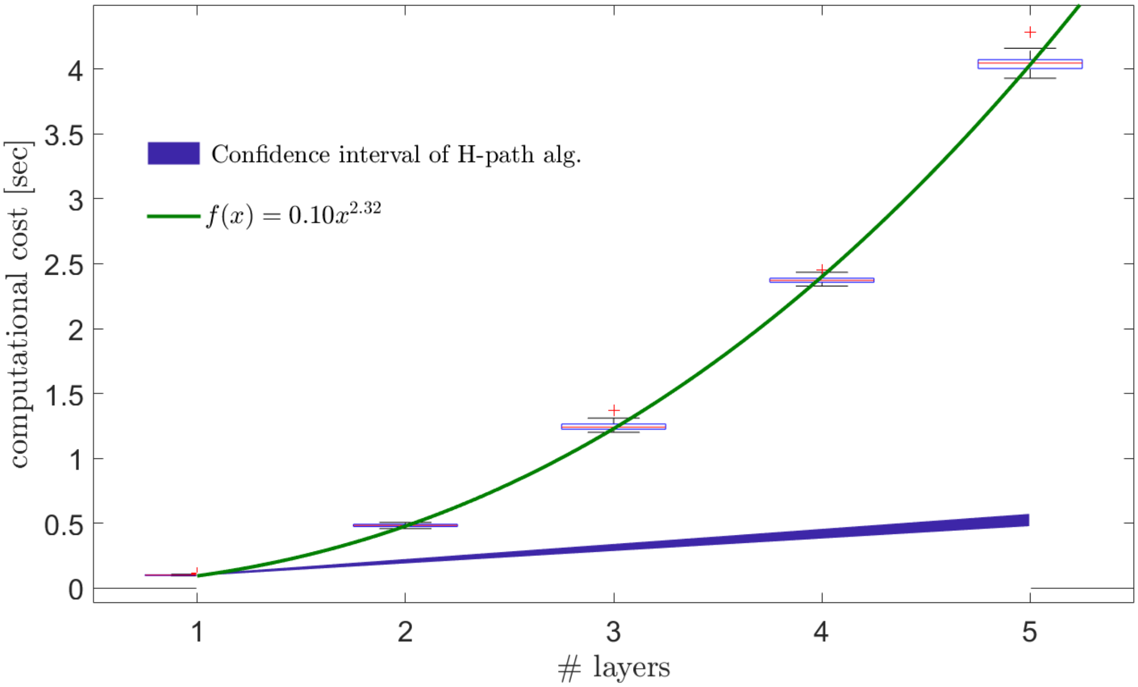

Fig. S4. Comparison between the network average efficiency with the network average speed. According to the formula, the average value of dynamical efficiency follows the average value of speed in the network. The main difference between these two measures comes from the spatial distribution among the link as highlighted in Fig. S5 and S6.

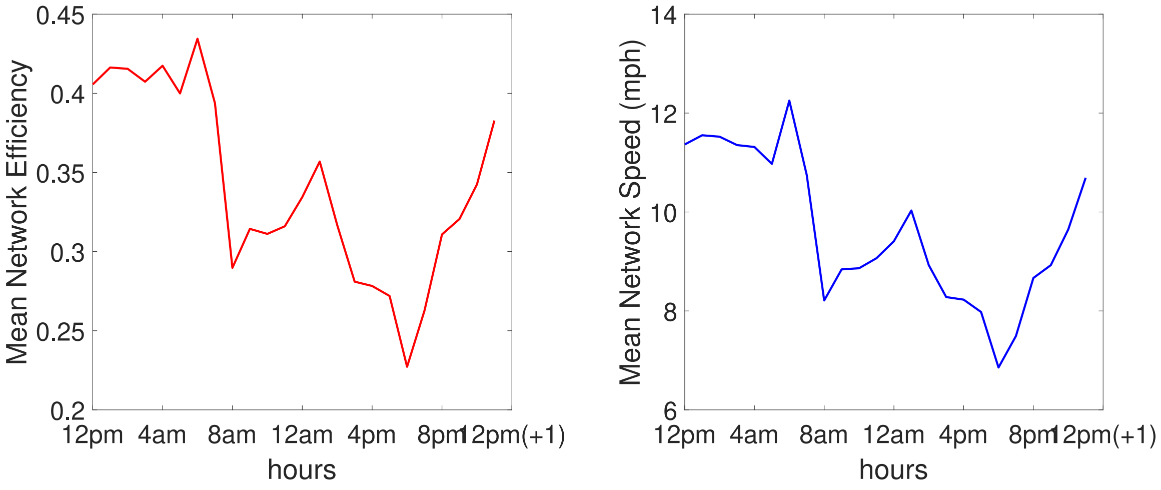

Fig. S5. Here is reported the map of the road efficiency for Shenzhen every 2 hours form 2am to 12 am. We can appreciate the emerging pattern of congestion that develops and propagates from the city center during the two peak hours. It reaches its lowest value around 8pm. The difference between the efficiency and the speed map (Fig. S6) it becomes, here, clear: the efficiency values are naturally smoothed and delimit the congested zones. The physical meaning of the dynamical efficiency expresses the concept of reachability of that specific location.

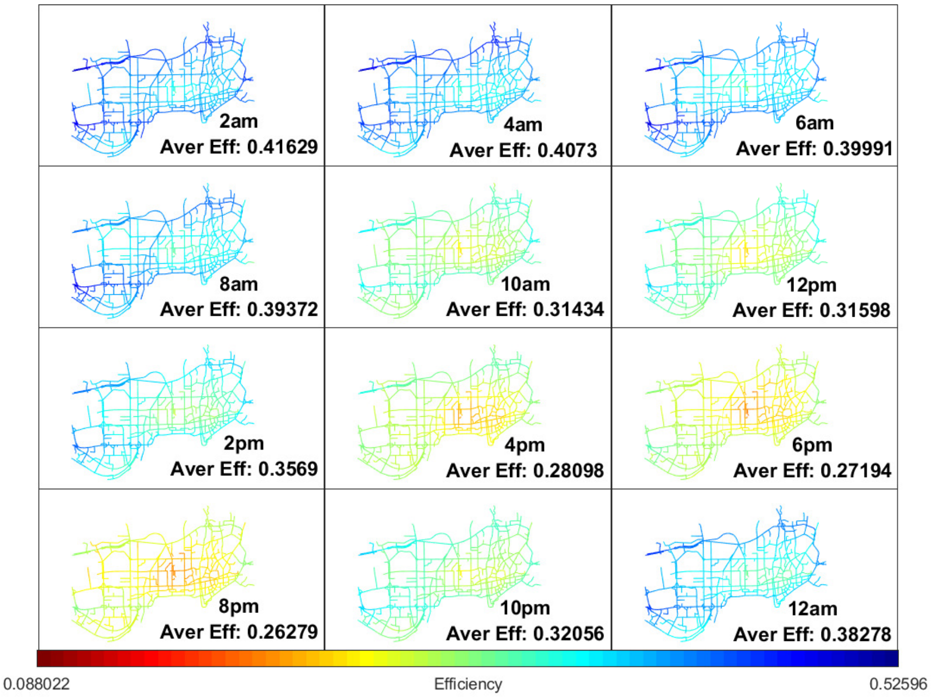

Fig. S6. Speed map of Shenzhen during the whole day. The same data have been used to calculate the efficiency in Fig. S5. We notice that in the speed representation of the map it is more difficult to separate the congested zones and the values (corresponding colors) are not spatially smoothed as in Fig. S5.

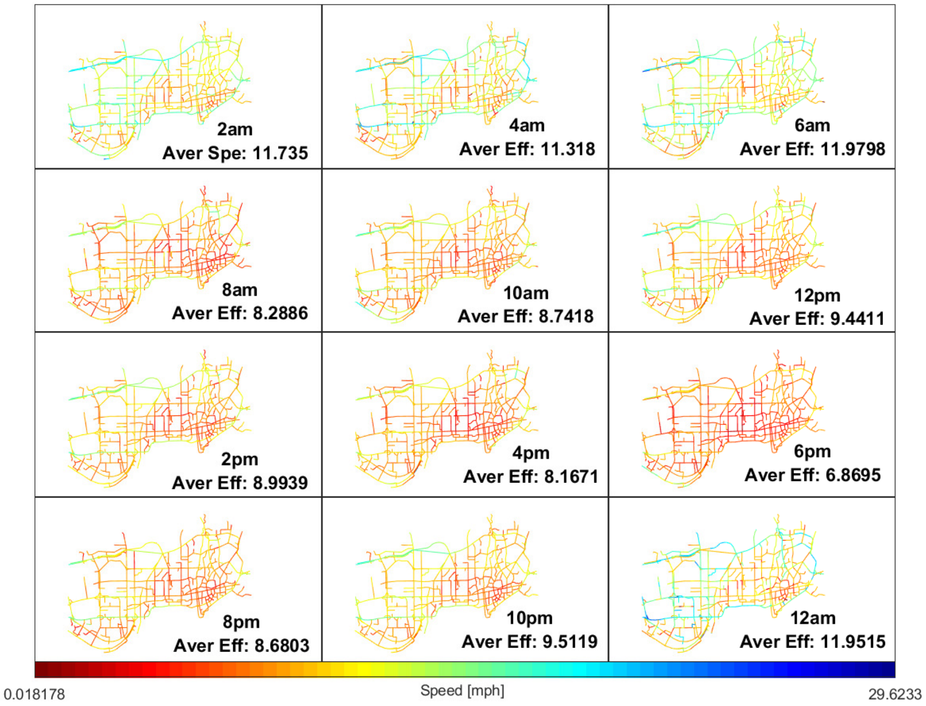

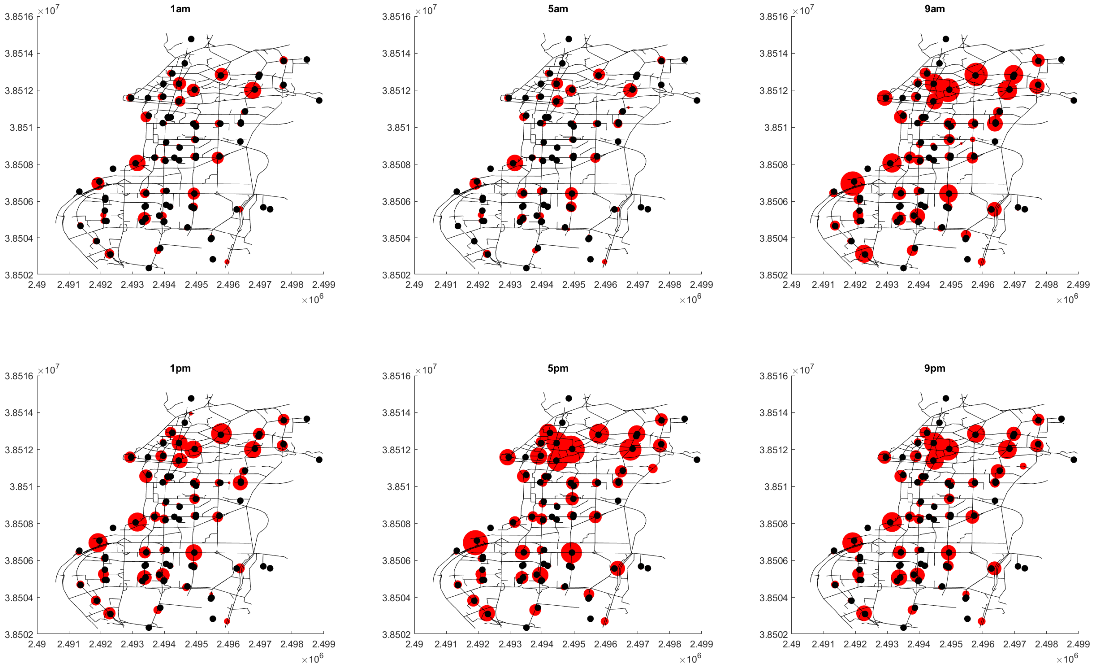

Fig. S7. Local α-dilemma factor during the day. Here it is represented the α dilemma factor, with $\alpha = 5\%$, for each interchange station. The red spot is proportional to the number of alternatives of that location if we allow the travel time to be until 5% more than the H-shortest path (that changes in k). We notice how the congestion, namely in the morning and evening peak hour, increases also the number of 'close' alternatives.

Fig. S8. Analysis of the data of trips.

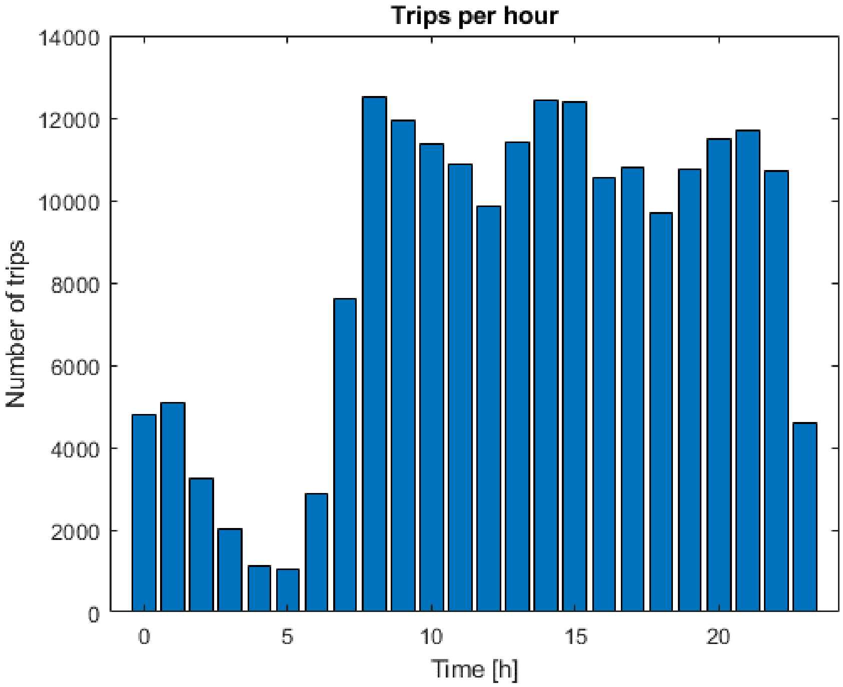

(a) Histogram of the number of trips extracted from our dataset in SHenzhen the 7th of September. The average is about 10000 per hour, with a maximum of 12491 recorded trips during peak hours (8 am) and the minimum 1043 at 5 am. In total about 220'000 trips.

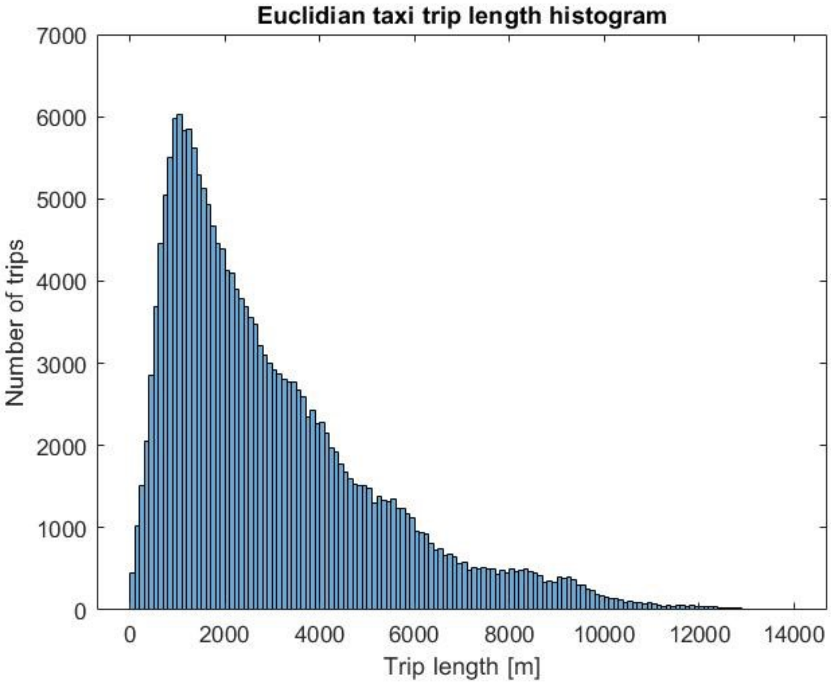

(b) Average trip length = 3013.5 m. Median = 2379 m

Fig. S9. Origin-destination zones.

(a) The division of the map of downtown of Shenzhen in 50 homogeneous rectangular zones. Every origin and destination of all taxis trips in our dataset has been assigned to its relative zone. In this way we were able to reconstruct the circular graph in Fig. S9b of the OD demand.

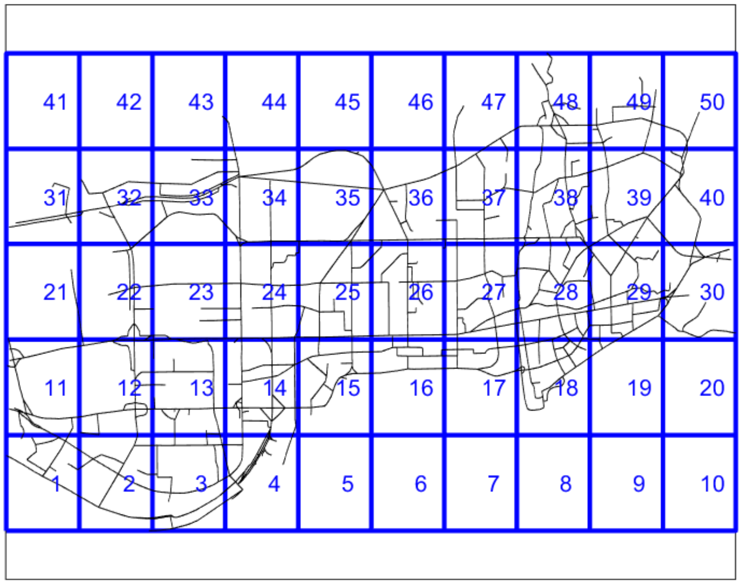

(b) Circular graph of demand among the 50 zones in Shenzhen downtown. The color and the thickness are proportional to the amount of registered trips during a whole day (7th September 2011).

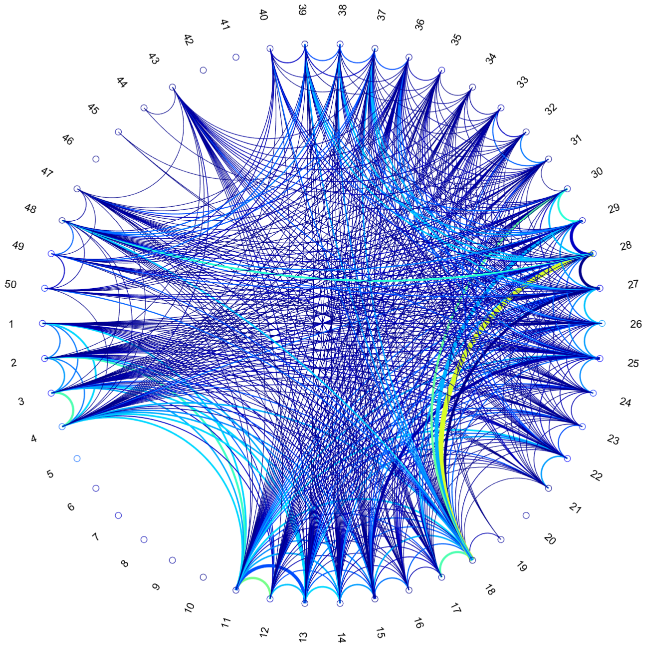

Fig. S10. Efficiency variation.

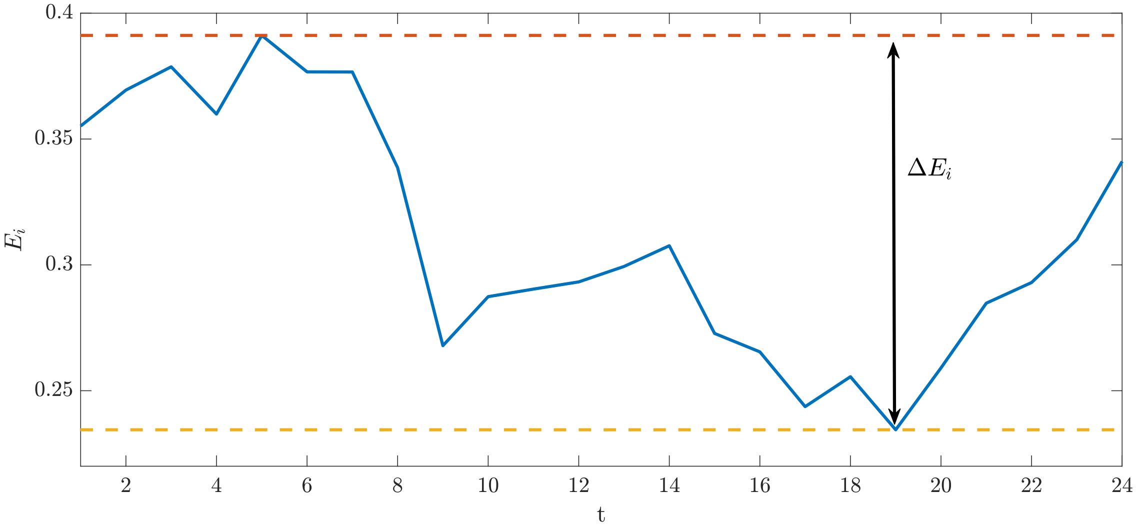

(a) Here the example of a single link efficiency E_i during the whole day. We define *daily efficiency variation* for each link as $\Delta E_i = \max_{t\in T} E_i(t) - \min_{t\in T} E_i(t)$, where $T = [1, 24]$ is a whole day. The daily variation, that corresponds to the maximum difference of single link dynamical efficiency is a measure of the variability of the network due to urban congestion pattern dynamics.

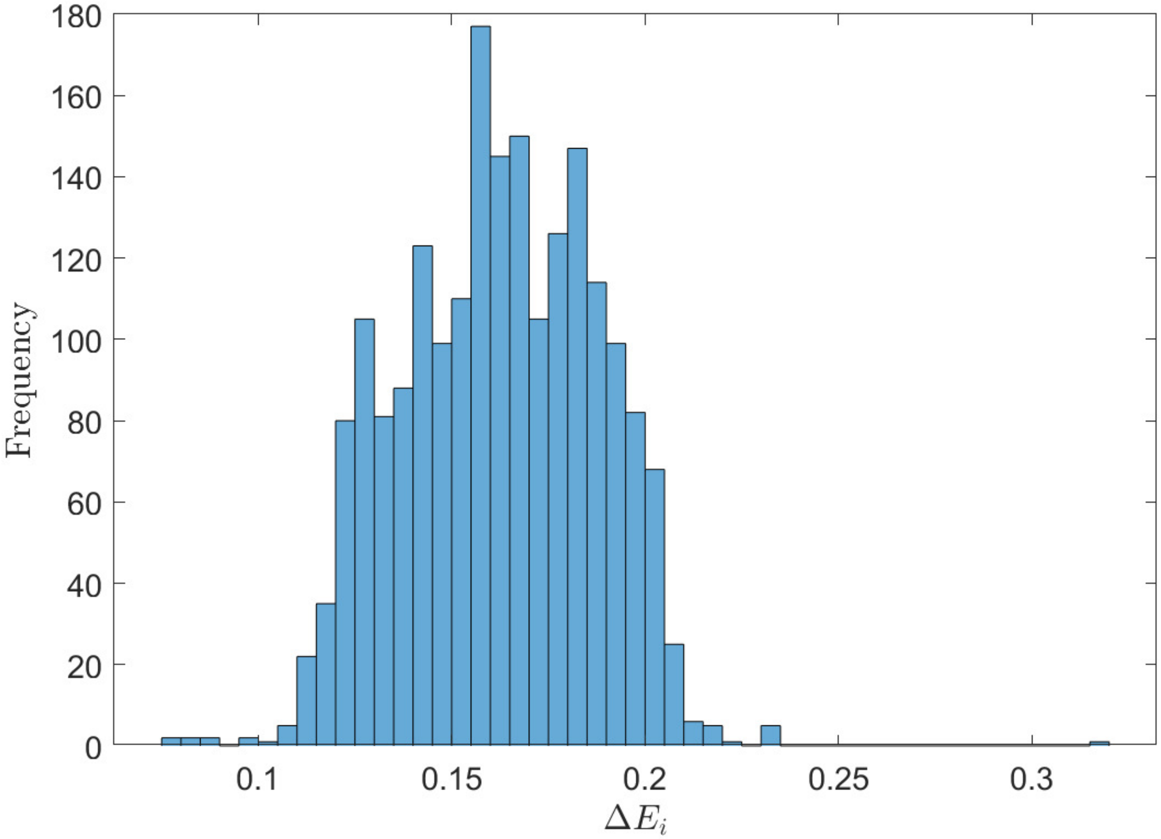

(b) The histogram of the daily efficiency variation ΔE_i of all link $i \in N$.

Fig. S11. Bike-sharing layer

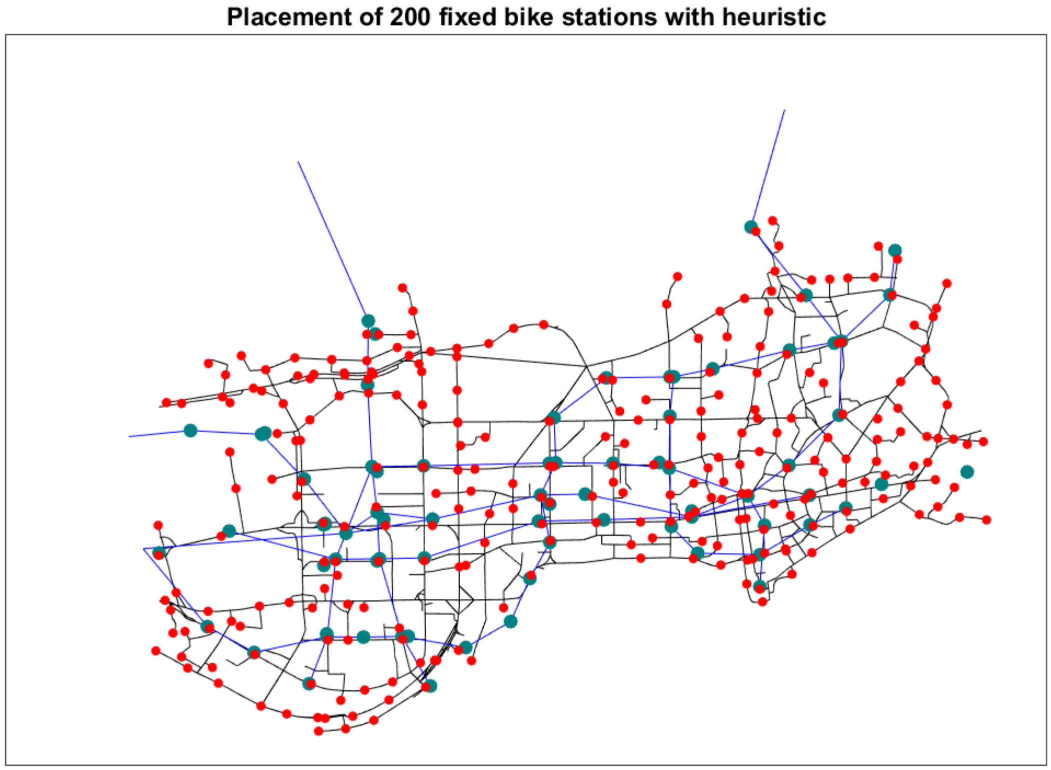

(a) Map of the location of the bike stations (red points) and the metro stations (blue points).The walking distance between 2 stations is always up to 5 minutes and the placement started from the less efficient links to improve the homogeneity in dynamical efficiency in the urban multi-layered network.

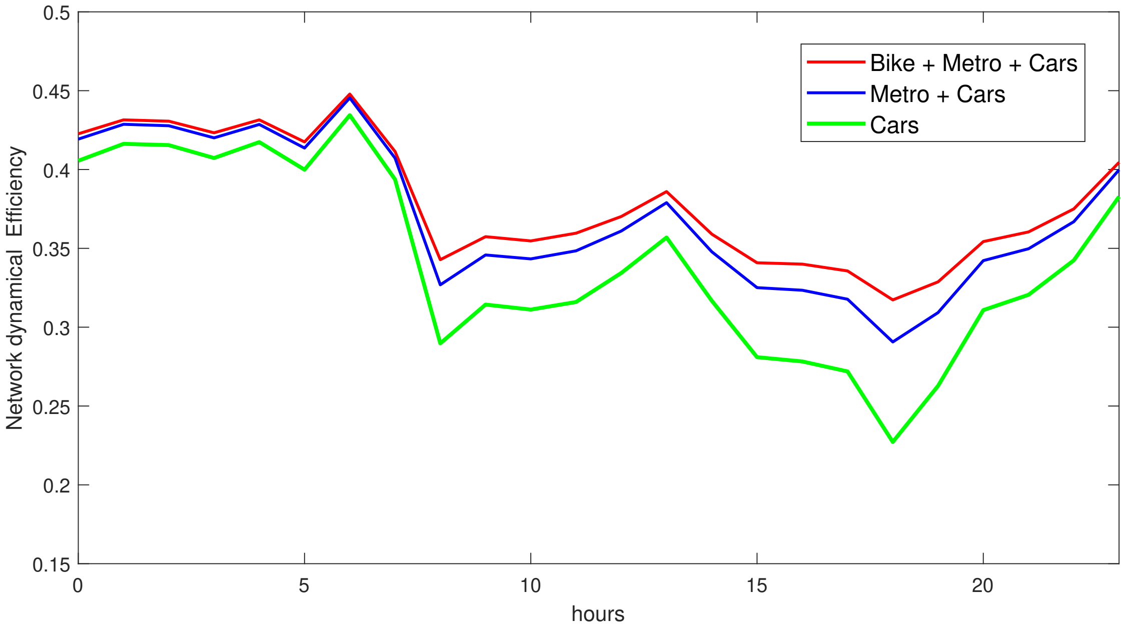

(b) Comparison during the whole day of network dynamical efficiency for the three different multi-layered networks: only car (in green), metro and car (in blue), car/metro and bike-sharing (in red).

Fig. S12. Improvements with the bike layer.

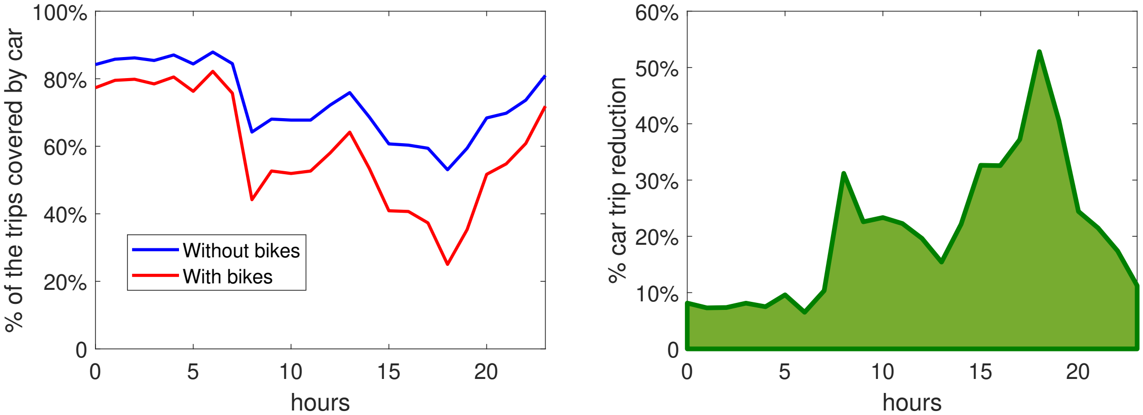

(a) In a scenario with 200 bike stations, distributed through the network of the city of Shenzhen with the heuristic explain in the text, we report the percentage of the trip covered by private cars in a all shortest H-path algorithm. On the panel on the right we visualized the car trip reduction due to the facilities of bike mobility.

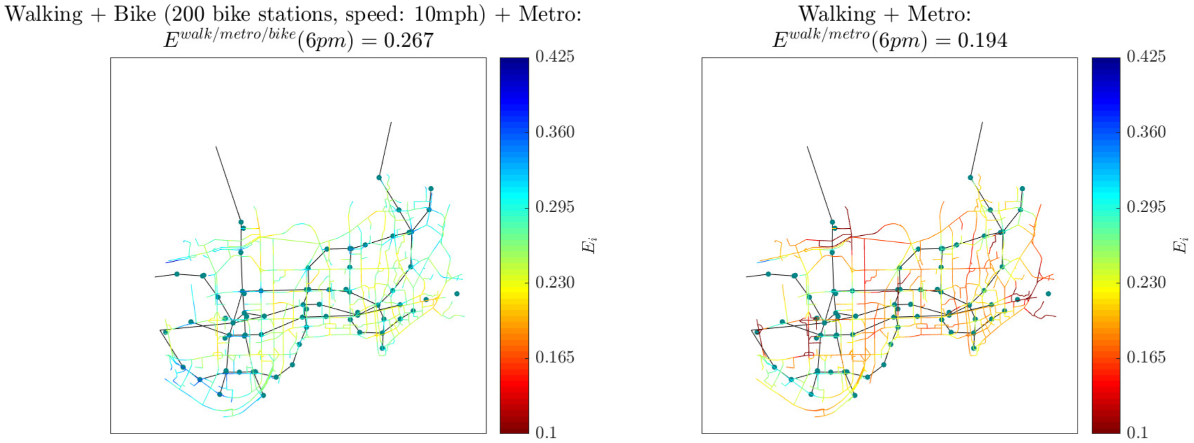

(b) Comparison of the color map of Shenzhen network for the link dynamical efficiency in a *walk/metro/bike* scenario and (on the left) in a *walk/metro* public transportation scenario. We can here appreciate the differences in link dynamical efficiency that a new mode of transportation, with the disposition of the intermodal junctions as described in the text, can change the configuration of the local pattern of dynamical efficiency and in particular have a more homogeneous distribution of transportation services.

Fig. S13. Statistics of dynamical efficiency after network percolation

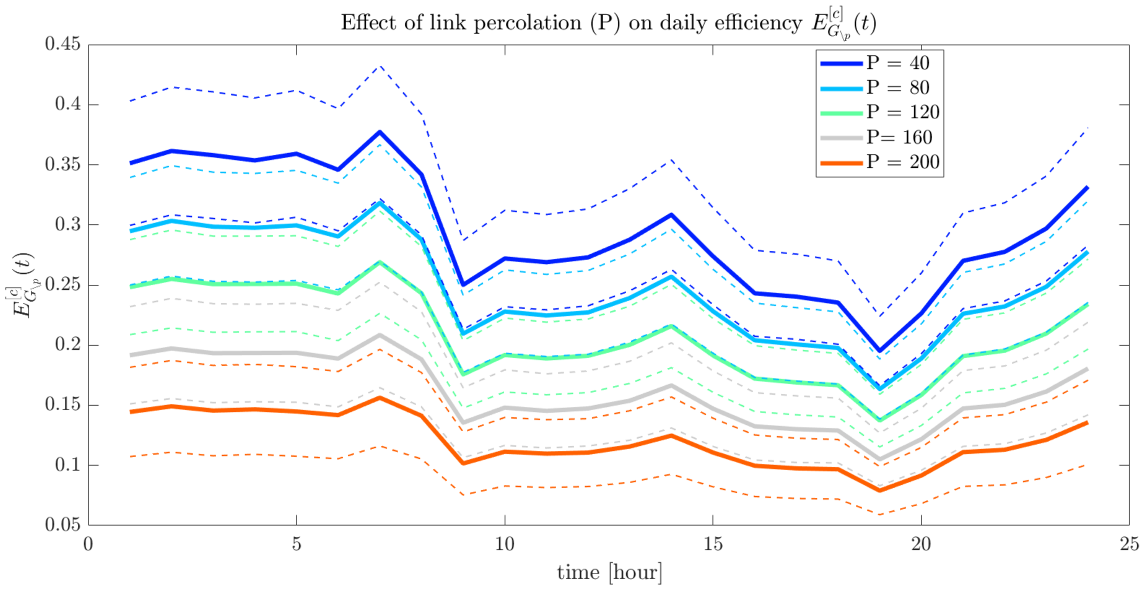

(a) In continuous line the mean of the network dynamical efficiency for $P = 40, 80, 120, 160, 200$ number of random links removed. In dashed line the standard deviation for the 50 simulated cases.

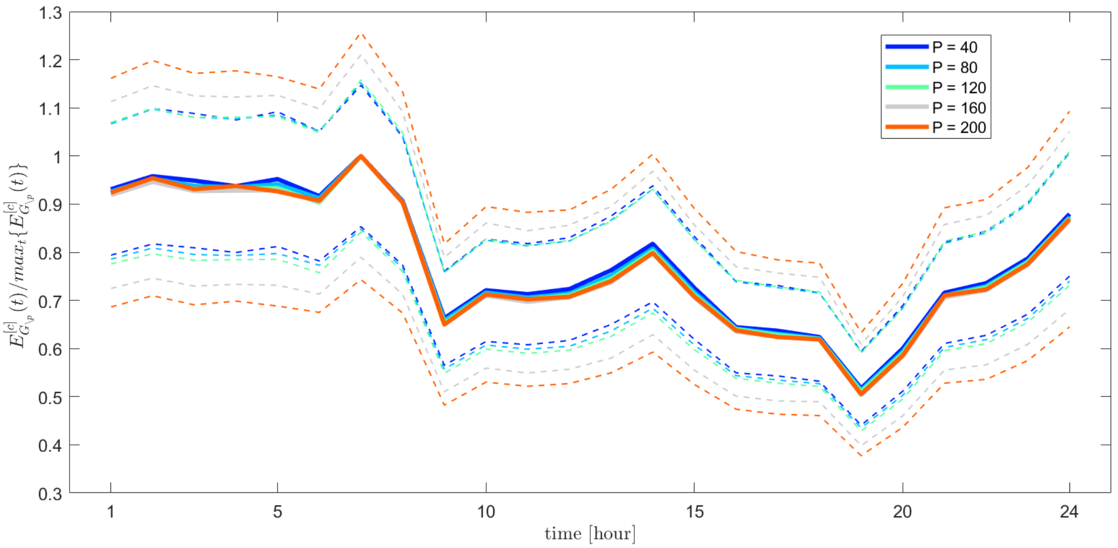

(b) Here the curves reported in Figure S13a have been divided by the corresponding maximum average value for each case P = 40, . . . , 200 (continuous lines in Fig S13a). The high similarity of the average value of the dynamical efficiency normalized by the corresponding max value supports the proof of the robustness of this measure respect to percolation and network variations. We also notice how the standard deviation among the tested 50 scenarios per case (P) tend to increase with P.

Fig. S14. Maximum and minimum value of dynamic efficiency over a day for different values of $P = 40, 80, 120, 160, 200$

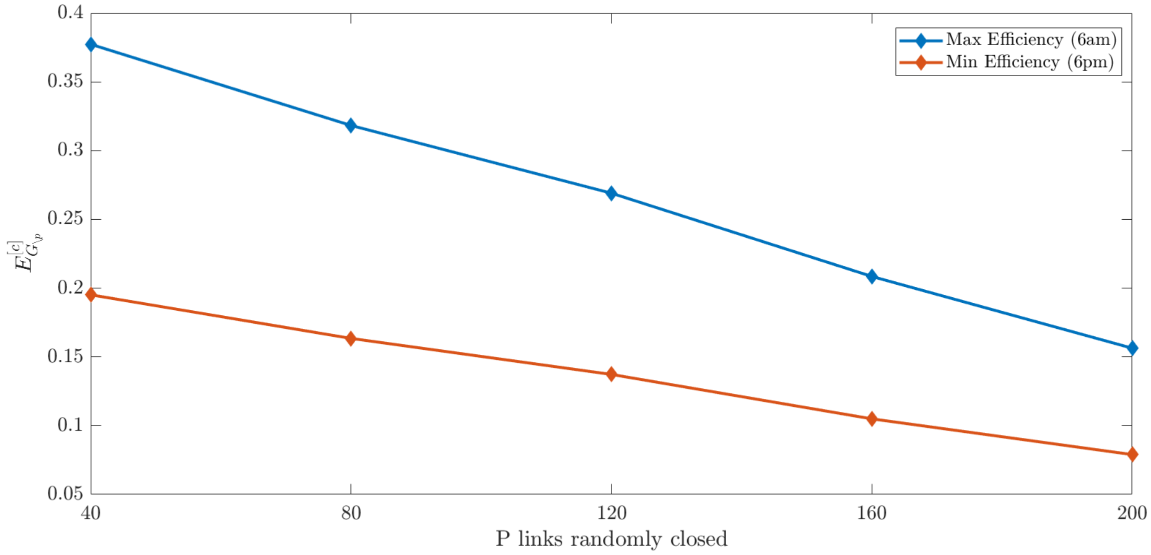

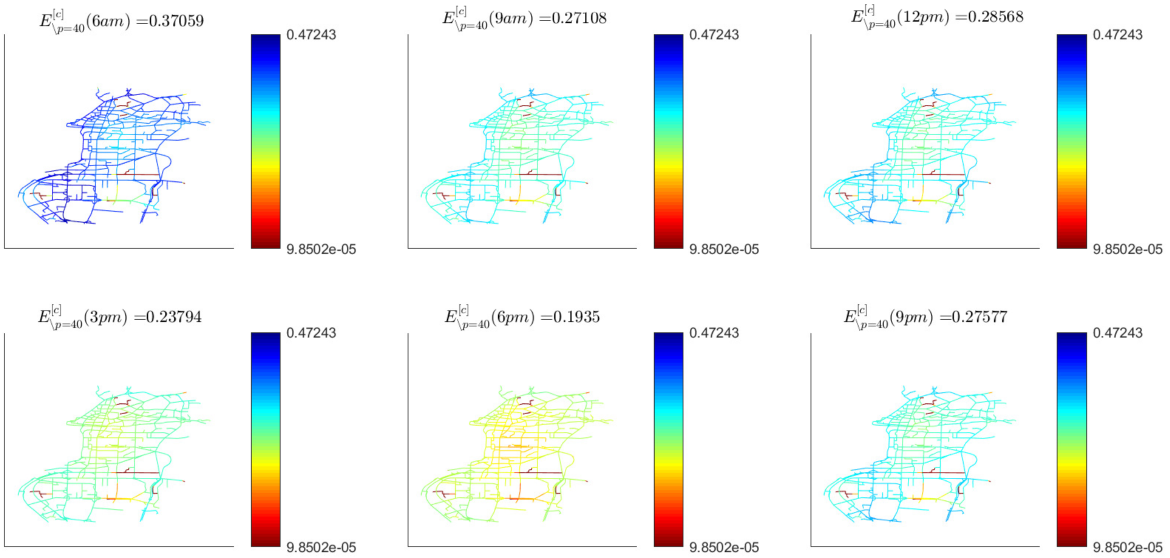

Fig. S15. Dynamical efficiency patter after link removal with $p = 40$ at $t =$ 6am,9am,12pm,3pm, 6pm, 9pm. The pattern here is very similar to the complete Shenzhen's graph used in the results section of the main paper. This is just one of the 50 cases used to deduce the statistics about robustness of dynamical efficiency with 40 links randomly removed (highlighted in dark red). We notice that the minimal value in the scale is very small but not zero. This is due to the fact that at least one extreme of each eliminated link is reachable by some path and, in this particular case, there is no isolated node.

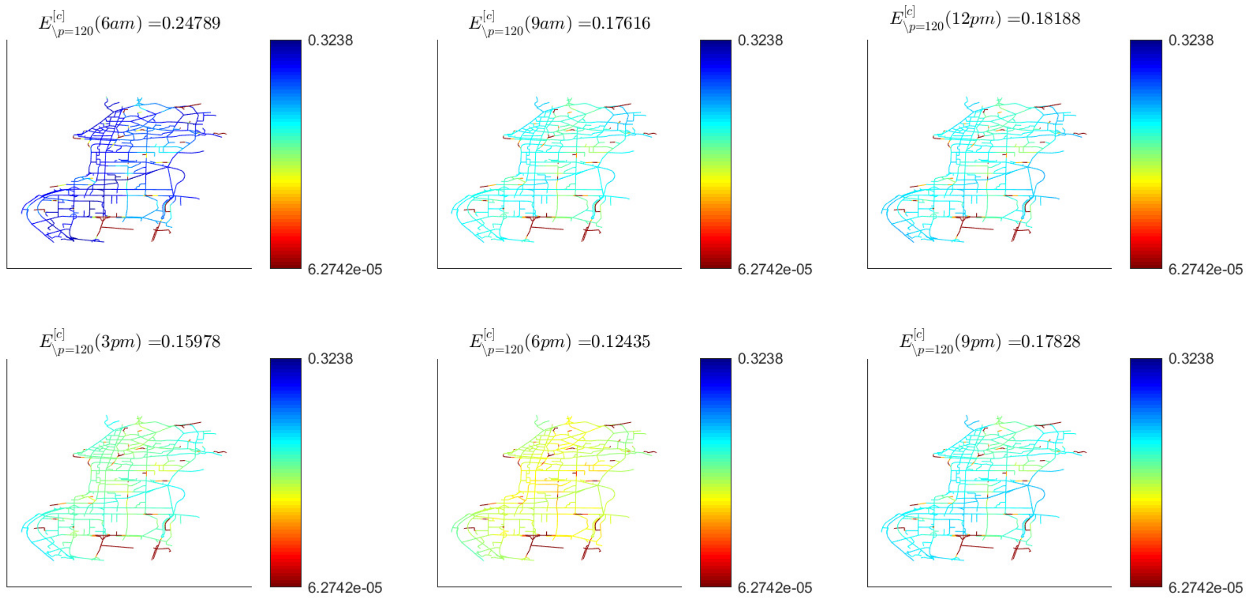

Fig. S16. Dynamical efficiency patter after link removal with $p = 120$ at $t =$ 6am,9am,12pm,3pm, 6pm, 9pm. In this case we begin to appreciate some differences in the spatial distribution of dynamical efficiency with respect to the complete graph. The congested zones are less defined and 120 links removed from the networks create some inefficiency roughly homogeneously distributed in all the city network.

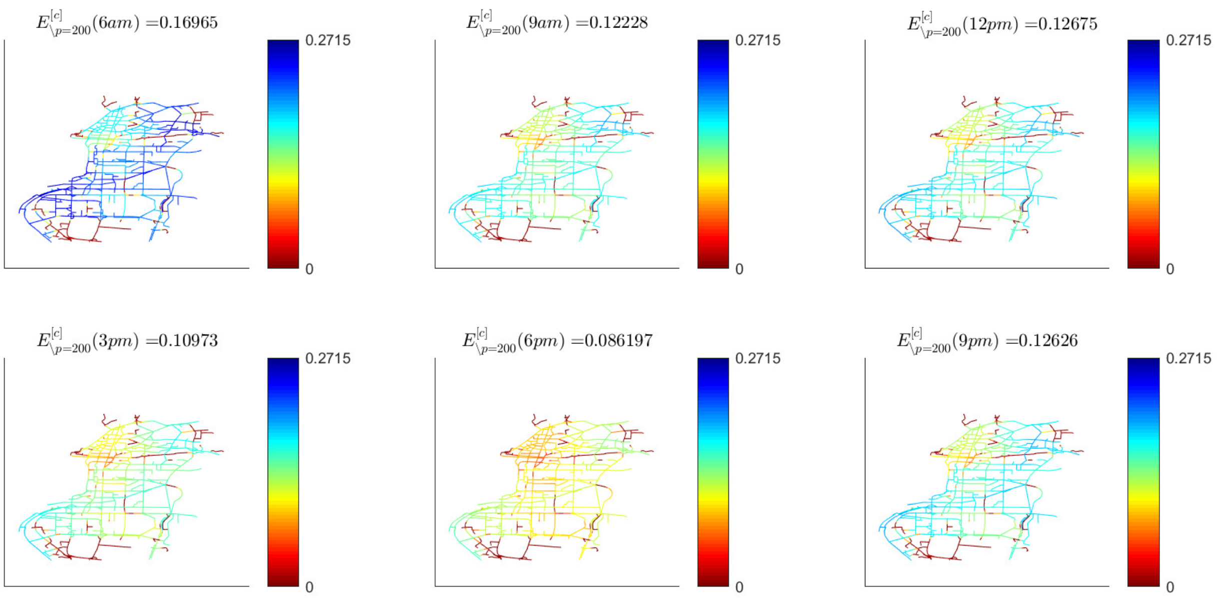

Fig. S17. Dynamical efficiency patter after link removal with $p = 200$ at $t =$ 6am,9am,12pm,3pm, 6pm, 9pm. In this particular scenario where 200 links ($\approx 10\%$ of the total network) has been removed from the network the spatial pattern of dynamical efficiency took a very different and defined configuration with respect to the complete graph. In particular, we notice a zone in the top-left part (instead of the top-central zone) of the map that has been heavily influenced by a few links removed around it. Also in the bottom part of the map, instead of the left-bottom part as in the complete graph, we have a right-bottom part considered as inefficient according the dynamical efficiency.
